# Zika virus impacts extracellular vesicle composition and cellular gene expression in macaque early gestation trophoblasts

**DOI:** 10.1038/s41598-022-11275-9

**Published:** 2022-05-05

**Authors:** Lindsey N. Block, Jenna Kropp Schmidt, Nicholas S. Keuler, Megan C. McKeon, Brittany D. Bowman, Gregory J. Wiepz, Thaddeus G. Golos

**Affiliations:** 1 Wisconsin National Primate Research Center, University of Wisconsin-Madison, 1223 Capitol Ct., Madison, WI 53715-1299 USA; 2 Department of Pathology and Laboratory Medicine, University of Wisconsin-Madison, Madison, WI USA; 3 Department of Statistics, University of Wisconsin-Madison, Madison, WI USA; 4 Department of Biomolecular Chemistry, University of Wisconsin-Madison, Madison, WI USA; 5 Department of Comparative Biosciences, University of Wisconsin-Madison, Madison, WI USA; 6 Department of Obstetrics and Gynecology, University of Wisconsin-Madison, Madison, WI USA; 7 Present Address: University of Pennsylvania, Philadelphia, PA USA; 8 Present Address: University of Nebraska Medical Center, Omaha, NE USA

**Keywords:** Cell biology, Mechanisms of disease, Infection

## Abstract

Zika virus (ZIKV) infection at the maternal–placental interface is associated with adverse pregnancy outcomes including fetal demise and pregnancy loss. To determine how infection impacts placental trophoblasts, we utilized rhesus macaque trophoblast stem cells (TSC) that can be differentiated into early gestation syncytiotrophoblasts (ST) and extravillous trophoblasts (EVT). TSCs and STs, but not EVTs, were highly permissive to productive infection with ZIKV strain DAK AR 41524. The impact of ZIKV on the cellular transcriptome showed that infection of TSCs and STs increased expression of immune related genes, including those involved in type I and type III interferon responses. ZIKV exposure altered extracellular vesicle (EV) mRNA, miRNA and protein cargo, including ZIKV proteins, regardless of productive infection. These findings suggest that early gestation macaque TSCs and STs are permissive to ZIKV infection, and that EV analysis may provide a foundation for identifying non-invasive biomarkers of placental infection in a highly translational model.

## Introduction

Maternal Zika virus (ZIKV) infection is associated with adverse pregnancy outcomes including pregnancy loss and fetal malformations^1,2^. Prolonged maternal viremia in humans and nonhuman primates^2–7^ suggests that there is a pregnancy-specific viral reservoir, and the presence of virus at the maternal fetal interface^8^ support the premise that the placenta may serve as that reservoir. Thus, there is a pressing need to better understand the mechanisms of placental infection and ZIKV impact on placental cell function.

The placenta is an essential organ in pregnancy as it not only conveys sufficient nutrients and oxygen for proper fetal development^9^, but also provides signals for the adaptation of maternal physiological systems to pregnancy. Errors in placental development and function are often associated with adverse pregnancy outcomes^9^. The placenta is comprised of several trophoblast cell types, including villous cytotrophoblasts (vCTBs), syncytiotrophoblasts (STs), and extravillous trophoblasts (EVTs), as well as fetal macrophages (Hofbauer cells), other immune and vascular cells, and fibroblasts^10^. vCTBs can be programmed to a proliferative stem cell-like state in vitro, termed trophoblast stem cells (TSCs) that can be differentiated into STs or EVTs^10–12^.

Maternal ZIKV infection early in pregnancy is associated with increased risk for pregnancy loss and more severe complications^2,4,13–17^. However, the ability of clinicians and investigators to assess the risk of vertical transmission is hindered by uncertainty in the timing of infection and the inability to directly assess placental, fetal, or reproductive tract tissues during pregnancy. In addition, in vitro studies^18,19^ have demonstrated that early gestation trophoblasts are permissive to ZIKV infection; however, the specific impact of infection on trophoblasts has not been comprehensively characterized. Inconsistencies in reports of trophoblast infection in vivo warrant further study of trophoblast-type permissiveness to ZIKV infection in early pregnancy to enable future development of methods to non-invasively monitor in vivo placental infection. Extracellular vesicles (EVs) are widely studied as a minimally invasive “liquid biopsy” to monitor cell function from in vivo body fluids or in vitro culture media^20,21^. EVs contain cell-type specific markers, which can be used to monitor the status of those cells^20^. The cargo packaged within EVs, including nucleic acids and proteins, is reportedly altered under diseased and infected states^22–24^.

Macaques are relevant models of human pregnancy^25^ and ZIKV infection during pregnancy^26–28^. In the current study, a rhesus macaque TSC model was utilized as it allows for the study of trophoblast cell type-specific responses. Macaque TSCs derived from first trimester vCTB maintain proliferation and can be directed to ST- or EVT-specific differentiation^12^. We showed previously that TSCs that have been differentiated to ST display features characteristic of the early first-trimester, a developmentally critical period before the definitive placenta has completely formed^12^. The objectives of this study were to (1) determine which macaque trophoblast cell types were permissive to ZIKV infection, (2) determine the molecular and secretory impact of ZIKV infection, and (3) determine the utility of placental EVs (PEVs) to serve as a readout of trophoblast infection status. The cellular and EV responses associated with infection presented here provide insight into how ZIKV impacts trophoblasts in the first trimester and suggests that PEV cargo may serve as a readout of placental ZIKV infection.

## Results

### Differentiation of TSCs alters cell permissiveness to ZIKV

**Figure 1:**
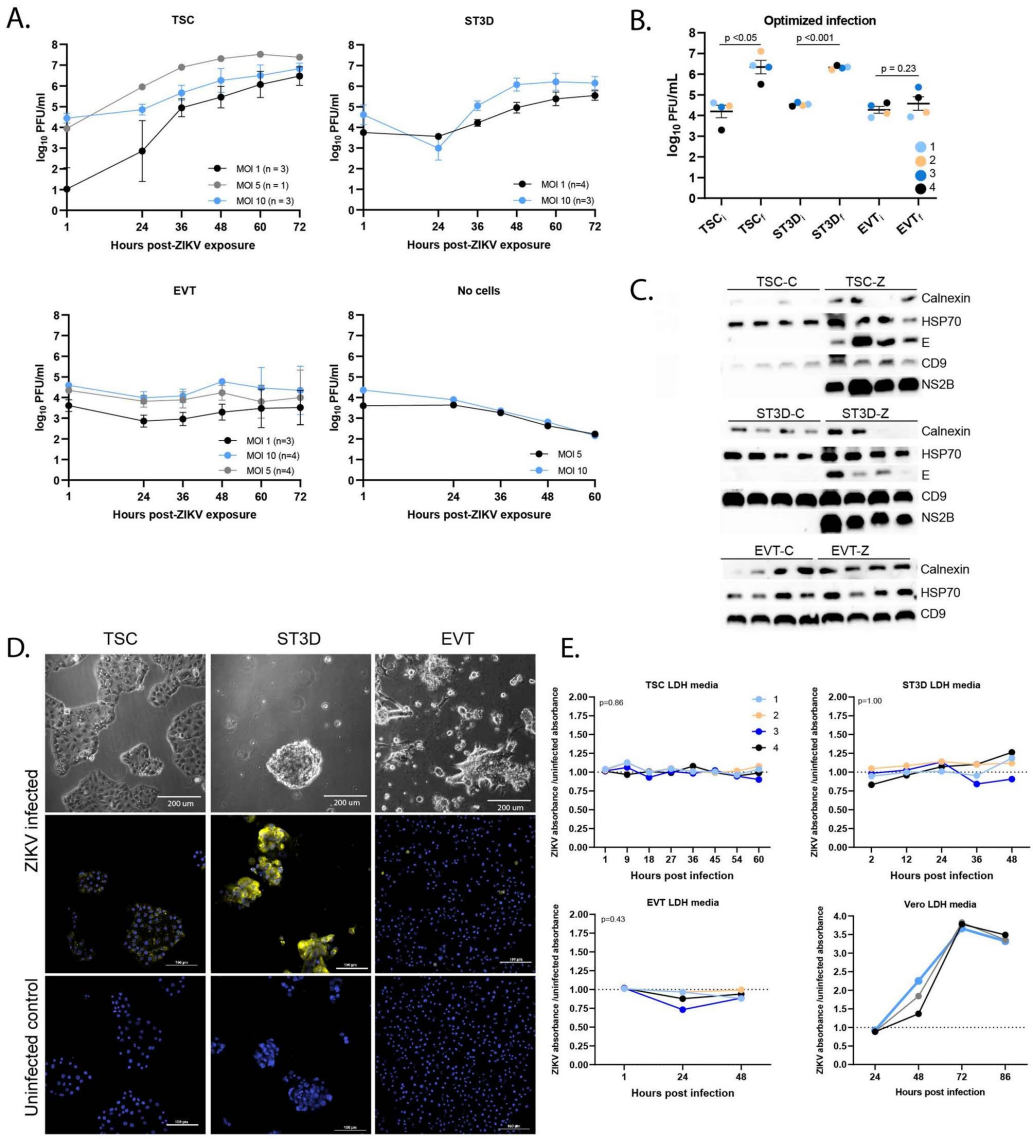
Early gestation trophoblast cells are permissive to ZIKV infection. (**A**) Quantification of infectious virus by plaque assay in a time course and multiplicity of infection (MOI) dose response for each cell type (TSC, ST3D, EVT, or “no cells”). The number of cell lines tested, n, is indicated within parentheses next to the MOI. The quantity of infectious virus detected at each time point represents accumulated virus over time since the previous time point from the same well. (**B**) Quantification of infectious virus by plaque assay with optimized MOI and duration of culture (i subscript = initial time point; f subscript = final time point) for each cell type and cell line (indicated by circle color). The mean plaque forming units (PFUs) ± the standard error of the mean (SEM) is shown. A paired *t* test with Bonferroni correction was used to determine significance. (**C**) Western Blots of ZIKV E, NS2B, CD9, Calnexin, and Heat shock protein 70 (HSP70; loading control) proteins for each cell line (n = 4) and cell type. (**D**) Immunostaining for ZIKV E protein. The top panel shows bright field images of trophoblasts after ZIKV exposure. ZIKV infected cells (middle panels) and control cells (bottom panels) were stained with an antibody against ZIKV E protein (yellow) and a nuclear stain (DAPI; blue). (**E**) Time course of LDH release by TSCs, ST3Ds, EVTs and Vero cells. Trophoblast data is presented as the mean of biological (n = 2–3) and technical replicates at each time point for the four indicated cell lines. The y-axis is presented as ZIKV LDH absorbance/control LDH absorbance, where a value > 1 indicates more cell death in ZIKV exposed cells and a value < 1 indicates less cell death in ZIKV exposed cells. The graph for Vero cell infection represents MOIs of 1 (black), 5 (grey) and 10 (blue). A paired *t* test was used to determine significance, which is indicated on the graphs.

Since maternal ZIKV infection early in pregnancy is associated with a high rate of pregnancy loss and fetal malformations, an early gestation in vitro macaque TSC model was used to determine which trophoblast cell types are permissive to infection. In vivo macaque^29^, mouse^30,31^, and porcine^32^ studies and in vitro human studies^19,33,34^ suggest that strains of the African lineage may be more detrimental to pregnancy than Asian lineage strains, hence, an African lineage strain was utilized to inoculate macaque TSCs. Preliminary time-course experiments to test multiplicities of infection (MOIs) were performed for each cell type to assess viral replication through 72 h of culture (Fig. 1A). The amount of infectious virus detected in conditioned media from TSCs and STs grown in suspension (ST3Ds) plateaued at 60 and 48 h, respectively. Inoculation with a MOI of 5 resulted in higher levels of infectious virus produced in TSCs compared to an MOI of 1 or 10, whereas viral replication was highest in ST3Ds with an MOI of 10.

An increase in infectious virus was not observed during the EVT time course. Since the quantity of virus did not decrease, to further verify viral replication, wells that did not contain cells but contained EVT culture extracellular matrix components (Matrigel and Col IV) (“no cells”) were exposed to virus and the media was evaluated by plaque assay. Compared to the amount of virus detected in the “no cells” wells at 60 h (mean 2.2 log_10_ plaque forming units (PFU)/ml), there was ~ 40-fold more virus (mean 3.8 log_10_ PFU/ml) in the EVT samples at 60 h (Fig. 1A). The infectious half-life of ZIKV is ~ 12 h^35^, which is consistent with viral replication and release of low levels of infectious virus in the EVT culture. For subsequent EVT infection experiments, cells were inoculated at an MOI of 5 and cultured for 72 h.

To minimize cytopathic effects and to maximally survey EVs, the length of culture was chosen based on when the amount of virus being produced began to plateau, expecting that EVs released during peak viral shedding would show the most impact. MOIs for the optimized infection experiments were chosen based on whether there was a substantial increase in viral production (see “Methods”). Of note, an initial (i) aliquot of culture medium was collected after ZIKV inoculation or mock inoculation, and then a final (f) aliquot of medium was collected at the culture endpoint to assess viral replication by plaque assay (Supplementary Fig. 1A). There was a significant increase in the amount of virus present after culture in the TSC and ST3D (p < 0.05 and p < 0.001, respectively, paired *t* test) (Fig. 1B). A minimal and nonsignificant change (p = 0.23) in infectious virus was detected in the EVT samples (EVTi = mean 4.28 log_10_ ± 0.14, EVT_f_ = mean 4.58 log_10_ ± 0.23; Fig. 1B) as anticipated from the data in Fig. 1A.

The presence of ZIKV E and NS2B proteins within inoculated and mock-inoculated trophoblasts was assessed to verify cellular infection. Both proteins were detected via Western Blot in the ZIKV exposed TSCs and ST3Ds but not in uninfected (control) cells (Fig. 1C). Neither ZIKV protein was detectable in inoculated EVT samples (Supplementary Fig. 2), supporting the minimal increase of infectious virus seen in plaque assays. Positive ZIKV E protein detection via immunofluorescent staining further supported that TSC and ST3D are clearly permissive to ZIKV, while few EVTs stained positive for ZIKV E. (Fig. 1D). Full Western Blots and isotype control immunofluorescence images are shown in Supplementary Figs. 2 and 3, respectively.

To determine if the MOIs chosen for inoculation induced a cytopathic effect, the quantity of LDH released into the media following inoculation was assayed in each cell type. Vero cells were used as a positive control as they are highly permissive to ZIKV, and the cell loss observed (Fig. 1E) was likely associated with cell death following infection. If ZIKV induced cell death, there would be more LDH secreted and therefore a ZIKV:Control ratio of greater than one. A ratio of greater than one was not consistently detected in trophoblast cultures, whereas an increase in the LDH ratio was observed in Vero cells by 48 h that continued through 86 h. To determine if the changes in LDH were significant, the LDH absorbances over the time course was averaged for each cell line and then a paired *t* test between the control average absorbance and ZIKV average absorbance was used. None of the changes in LDH were significant (TSC p = 0.86; ST3D p = 1.00; EVT p = 0.43). Due to the lack of biological replicates, statistical analysis on the Vero LDH data was not completed. However, based on the increased quantity of LDH detected in the media, the data support that ZIKV exposure induced cell death regardless of MOI (average LDH absorbance: MOI 1 = 1.8, MOI 5 = 1.9, MOI 10 = 1.9, uninfected = 0.7). These data indicate that the MOI and viral strain chosen for this study did not result in trophoblast cell death during the culture period as the changes in their LDH release were not significant.

### ZIKV infection impacts cellular innate immune gene expression

**Figure 2:**
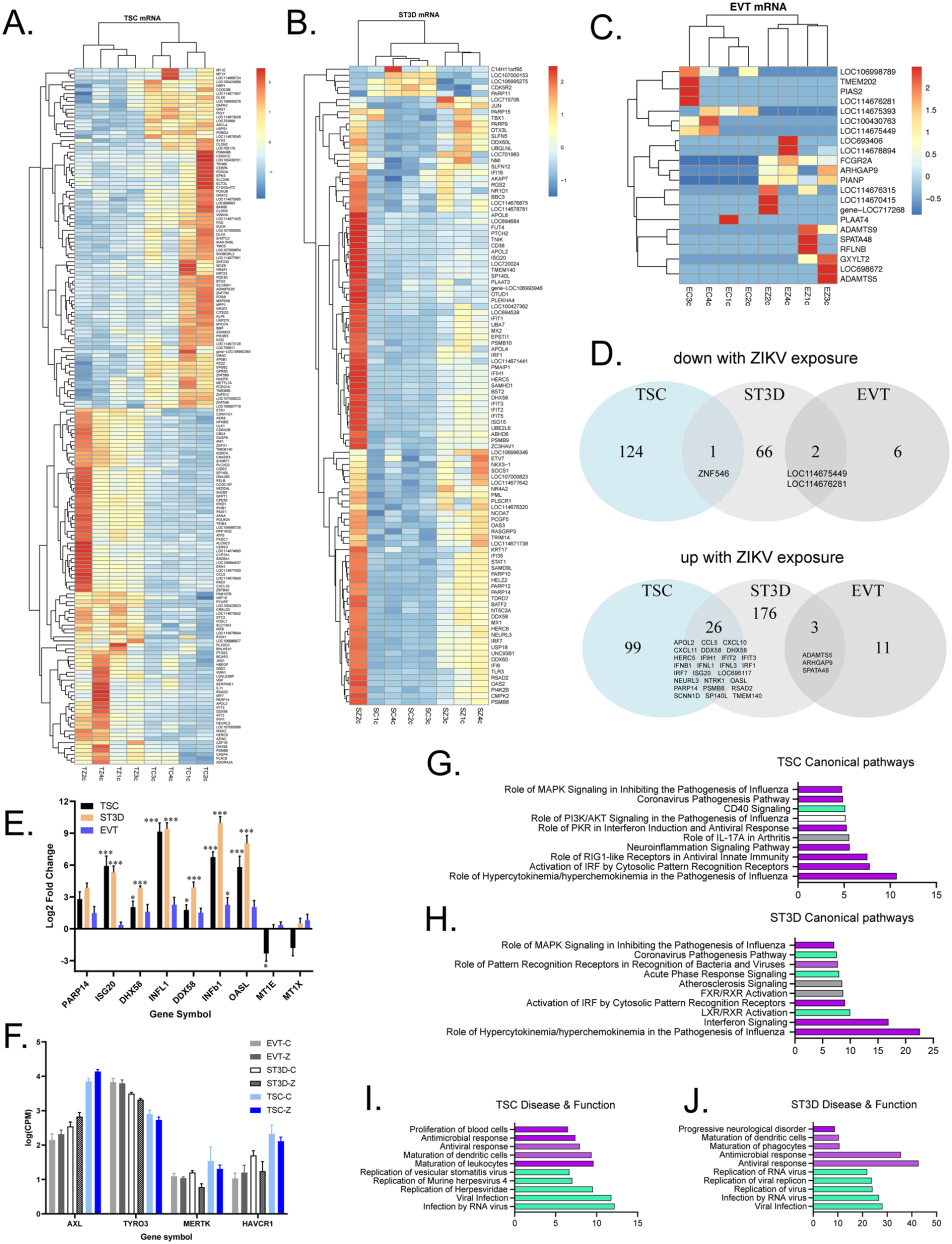
The impact of ZIKV on the cellular transcriptome. (**A**–**C**) Heatmaps of significantly differentially expressed mRNAs detected in all samples for TSCs and ST3Ds, and all significantly differentially expressed mRNAs in EVTs. (**D**) Venn diagrams of the number of genes downregulated and upregulated by ZIKV infection in TSCs, ST3Ds, and EVTs. (**E**) A bar graph of differential mRNA expression (log2 fold change) validated by qRT-PCR in which a value greater than 0 means more presence of that gene in ZIKV samples and a value less than 0 indicates decreased presence in ZIKV samples. A 2-way repeated measures ANOVA with Bonferroni correction was used to determine significance, which is depicted on the graphs (*p < 0.05; **p < 0.01; ***p < 0.001). Significance on the graph indicates whether that gene was significantly altered by ZIKV exposure compared to the respective cell type controls. Cell type comparisons can be found in Supplementary Table 4. (**F**) Normalized CPMs by DESeq2 for candidate ZIKV receptors. (**G**,**H**) Top 10 canonical pathways identified by IPA in the TSC and ST3D datasets [− log_10_ (p-value)]. Purple indicates a positive Z-score (activated in ZIKV); green indicates a negative Z-score (inhibited in ZIKV); white indicates 0 Z-score (no clear indication of whether the pathway is decreased or increased in ZIKV); and gray indicates unknown direction. (**I**,**J**) Top five predicted decreased (green) and increased (purple) disease and functions.

RNA sequencing was performed to assess the impact of ZIKV on the cellular transcriptome. Transcriptomic analysis by poly(A)seq identified 250 (125 upregulated, 125 downregulated), 274 (205 upregulated; 69 downregulated), and 22 (14 upregulated, 8 downregulated) differentially expressed genes (DEGs) in infected TSCs (TSC-Zs), ST3Ds (ST3D-Zs), and EVTs (EVT-Zs), respectively, compared to controls (Fig. 2A–C). The heatmaps in Fig. 2 depict only genes detected in every TSC (178 genes) or ST3D (105 genes) sample for ease of reading. Full heatmaps presented in Supplementary Fig. 4 show a similar clustering pattern when genes that were only detected in as few as one sample were included. The ST3D samples cluster fully by infection status when all genes were analyzed. TSC and EVT samples clustered by infection status, whereas one ST3D-Z sample clustered separately. Figure 2D presents Venn diagrams of significantly downregulated (top) and upregulated (bottom) mRNAs within the three trophoblast cell types. Transcriptomic analysis identified 26 upregulated genes in both TSC and ST3D, many of which were involved in the immune response.

The expression of seven of the genes involved in the antiviral response pathway (*PARP14, ISG20, DHX58, INFL1, DDX58, IFNB1,* and *OASL*) and two genes involved in the anti-apoptosis pathway (*MT1E* and *MT1X*)^36,37^ were validated with qRT-PCR (Fig. 2E; primer sequences Supplementary Table 2). The trends in expression observed with qRT-PCR agreed with those identified by poly(A)-seq analysis (Supplementary Table 4). TSC-Z and ST3D-Z samples had significantly increased expression in all seven genes, whereas significant elevation in *IFNL1, DDX58*, *IFNB1*, and *OASL* genes was detected in EVT-Z (Fig. 2E). A trend in decreased expression of *MT1E* and *MT1X* was confirmed in TSC-Z samples by qRT-PCR, supporting the RNAseq data. p-values are listed in Supplementary Table 5. There was no significant difference in PPAR14 expression with ZIKV exposure, but there was a difference between cell types (Supplementary Table 4).

Potential receptors for ZIKV entry were detected in the Poly(A)seq data, including *AXL, TYRO3, MERTK*, and *HAVCR1* (*TIM1*) (Fig. 2F)^19^. *DC-SIGN* was not detected. *AXL* and *HACVR1* expression were greatest in TSCs whereas TYRO3 expression was greatest in EVTs. No significant difference in receptor expression was observed between infected and control cells, in agreement with a previous report with human induced pluripotent stem cells (iPSCs)^38^.

Integrated Pathway Analysis (IPA) was performed on the TSC and ST3D datasets but could not be completed on EVT due to the limited number of DEGs. The top canonical pathway upregulated by ZIKV infection in TSC-Z and ST3D-Z cells was “role of hypercytokinemia/hypterchemokinemia in the pathogenesis of influenza” (Fig. 2G,H). Disease and Function IPA of the TSC-Z and ST3D-Z data predicts inhibition of the “infection by RNA virus”, “viral infection”, and various pathways associated with viral replication (Fig. 2I,J). Complementary to this finding, antimicrobial and antiviral responses were predicted to be activated in both TSC-Z and ST3D-Z.

### ZIKV infection altered the cellular miRNAome

**Figure 3:**
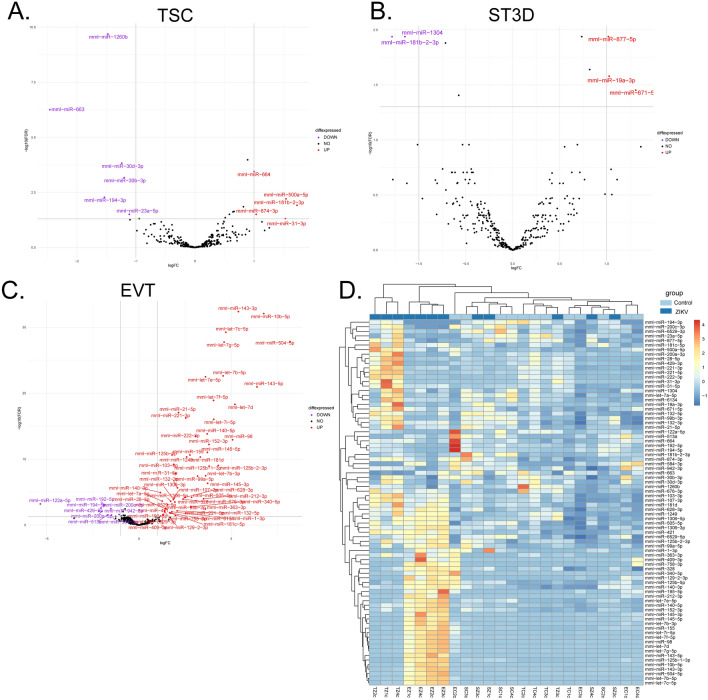
The impact of ZIKV exposure on the cellular miRNAome. Volcano plots of miRNA expression across for (**A**) TSC, (**B**) ST3D, and (**C**) EVT cells. Significantly differentially expressed miRNAs are displayed in color in the volcano plots for TSCs and EVTs (Purple indicates a decrease in ZIKV samples; Red indicates an increase; Black indicates no significant change). (**D**) A heatmap of all significantly differentially expressed miRNAs detected across the cell types.

Placental miRNAs are temporally expressed throughout gestation and have critical roles in trophoblast differentiation and function^39,40^, thus the miRNAome was profiled to assess alterations in expression relative to infection. miRNA-seq identified expression of 325 miRNAs in TSCs, 333 miRNAs in ST3Ds, and 323 miRNAs in EVTs. In the TSCs, six miRNAs were significantly decreased and five were significantly increased in infected versus control cells (Fig. 3A). Although ST3Ds were highly infected, only five miRNAs (two down, three up) were significantly impacted by ZIKV exposure (Fig. 3B). Conversely, despite modest productive infection of EVTs, 56 miRNAs were significantly increased and nine significantly decreased in infected versus control EVTs (Fig. 3C). To determine if ZIKV exposure resulted in similar trends of altered miRNA expression across trophoblast cell types, miRNAs significantly impacted in one cell type were compared to the other cell types. No miRNAs were significantly altered across cell types. However, samples tended to cluster by infection status (Fig. 3D). Interestingly, 12 miRNAs significantly impacted by ZIKV exposure were only detected in the EVT samples, which may be due to their differentiation or reaction to ZIKV.

### ZIKV infection did not alter trophoblast secretion of hormones and modestly impacted cytokine secretion

To assess the impact of infection on trophoblast function, secretion of hormones, cytokines, chemokines, and growth factors within conditioned cell culture media was assayed. Chorionic gonadotropin (CG) and progesterone are two key hormones in the recognition and maintenance of pregnancy. A significant difference was not detected in CG or progesterone secretion with ZIKV exposure, although there were significant cell type-dependent changes in CG secretion (TS vs ST3D, p < 0.001; TS vs EVT, p < 0.001; Supplementary Fig. 5). There also was a significant cell type-dependent change in progesterone secretion (TS vs ST3D, p < 0.01; TS vs EVT, p < 0.001). These findings support cellular differentiation from the TSC state and that this model is representative of the different placental cell types. TSCs secreted minimal CG or progesterone and ST3Ds and EVTs secreted significantly more CG than TSCs, in agreement with our previous report^12^.

Cytokines and growth factors associated with infection and the inflammatory response were quantified by Luminex assay (Supplementary Fig. 5B,C). *ITAC* (*CXCL11*) was significantly upregulated in infected ST3Ds compared to control (a similar trend was observed in *CXCL11* mRNA expression, Fig. 2B), and was more highly expressed in infected ST3Ds compared to infected TSCs. Otherwise, no other significant differences in cytokine or growth factor secretion were observed between infected and control cells. Regardless of infection status, there were significant differences in the secretion of several cytokines and growth factors between trophoblast cell types (Supplementary Fig. 5B). ST3D and EVTs expressed significantly more *IL-1RA* and *bNGF* than TSCs. *IL-6* was significantly upregulated in EVTs compared to ST3Ds. *FGF-2, VEGF-A, and VEGF-D* expression were significantly increased in ST3D cells compared to TSCs or EVTs. Conversely, significant differences were not detected across cell type or infection status (Supplementary Fig. 5C). p-values for hormones and analytes are listed in Supplementary Table 5.

### Characterization and impact of ZIKV infection on EVs

**Figure 4:**
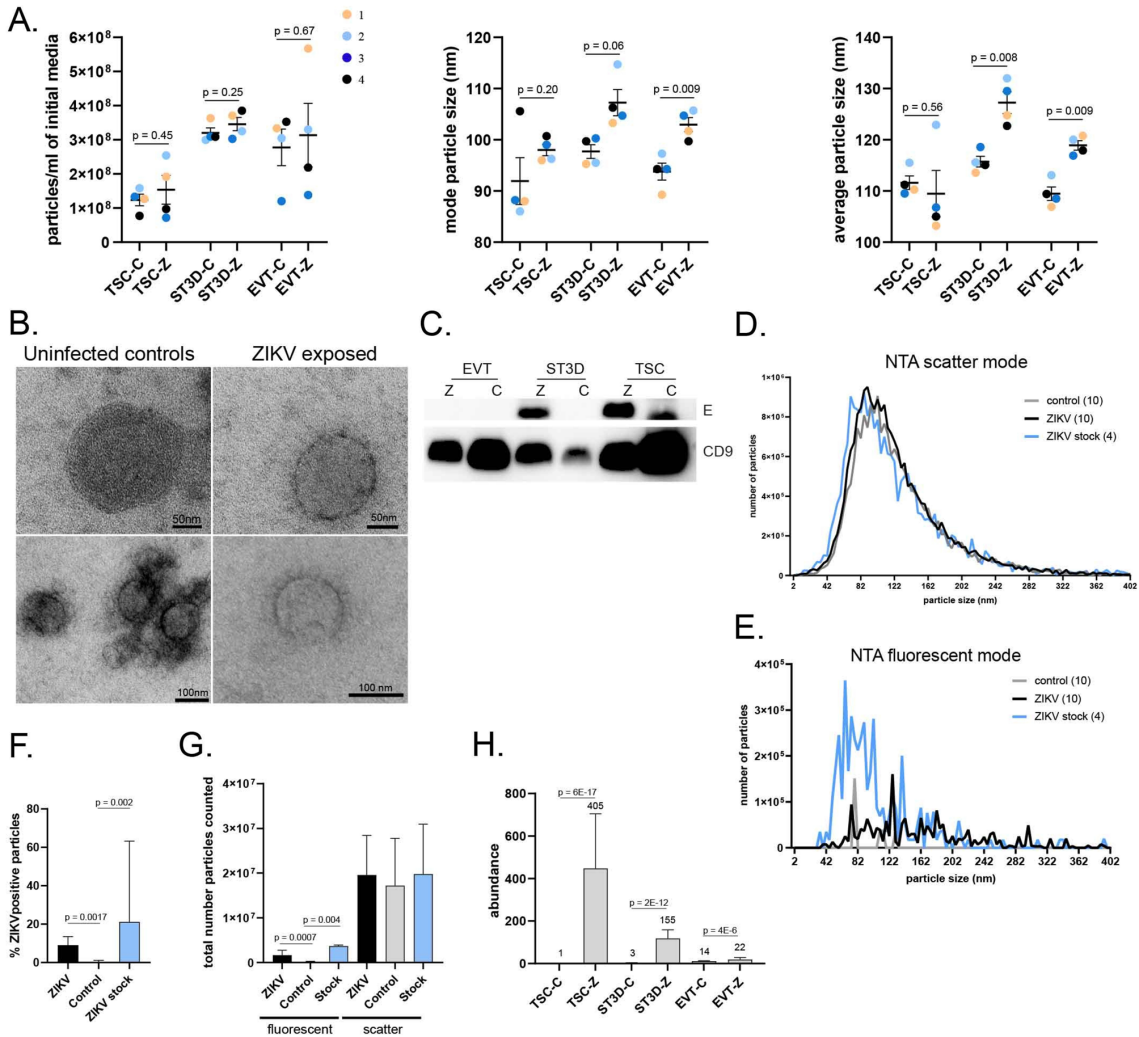
EV characterization from trophoblast-conditioned media. (**A**) NTA analysis of the particles presented as the particles/ml, the mode, and average particle size in nm for all 24 EV samples. Paired t-tests with Bonferroni correction were done to check significance. (**B**) Representative TEM images of four EV samples (two control and two ZIKV) with scale bars included. (**C**) Western blots of EV samples for ZIKV E and CD9. (**D**,**E**) EV samples were characterized with NTA scatter and fluorescent mode. The average number of particles detected across the control samples, ZIKV samples, and ZIKV stock were plotted against particle size. The number of samples is indicated in the parentheses. Bar graphs of the (**F**) percentage (%) of ZIKV positive particles and (**G**) the total number of particles counted. Graphs show the median and interquartile range. Statistical analysis was done with unpaired Mann–Whitney tests. (**H**) The average ZIKV protein abundance (numbers on the graphs) by group as determined by mass spectroscopy. Significance was determined with a *t* test (background based) and Benjamini–Hochberg correction (FDR). Significance is indicated on the graph where applicable.

EVs were isolated from ZIKV-inoculated and control trophoblast-conditioned media to assess changes in their physical properties and cargo following ZIKV exposure. EV samples were characterized by Zetaview NTA (Fig. 4A). Secreted particle concentration was variable and neither impacted by ZIKV nor significantly different across cell types, although Fig. 4A shows that TSCs tended to release fewer EVs than ST3Ds or EVTs. A consistent trend towards an increased mode particle size was observed in TSC-Z and ST3D-Z EVs compared to their controls (Fig. 4A), and the mode EVT-Z EV size was significantly larger than EVT-C EVs (p = 0.009). The average particle size also was significantly increased by ZIKV in ST3Ds and EVTs (p = 0.009), but not TSCs. TEM imaging verified the appropriate shape and size of isolated EVs (Fig. 4B).

To determine if ZIKV proteins were packaged into EVs, the E protein, which is highly abundant in mature virions and present in the secretory pathway^41^, was assessed by Western Blot. The ZIKV E protein was readily detected in the TSC-Z and ST3D-Z EV samples with none detected in the EVT-Z EV samples or any controls (Fig. 4C; Supplementary Fig. 3D). Since EVTs were only modestly infected with ZIKV, it is not surprising that the E protein was not detected. A nonspecific band of smaller size appeared in the TSC-C sample. The presence of CD9, a protein commonly enriched in EVs, also was observed.

To more directly assess whether the ZIKV E protein was associated with EVs, three to four samples from each control and infected cell type were stained with a fluorescently labeled ZIKV E antibody and analyzed by Zetaview NTA. EV preparations and concentrated ZIKV stock (positive control) were analyzed under scatter and fluorescent modes (Fig. 4D,E, respectively). A greater proportion (p = 0.08) of particles stained positively for the ZIKV E protein in the ZIKV stock (avg 31.5%, range 9.5–74%) compared to ZIKV exposed samples (avg 9.6%, range 0–20%) (Fig. 4F). As expected, the ZIKV stock and ZIKV exposed EVs contained significantly more positive particles compared to controls (avg 1.6%; range 0–11.3%; stock vs control p = 0.002, ZIKV exposed vs control p = 0.0017; Fig. 4F). While positive staining was detected in 2 out of 10 control samples, this is most likely due to antibody aggregation or some non-specific binding; only 1 and 3 actual particles were detected by the Zetaview, which then used an algorithm to calculate total particles/ml. A significant difference was not detected between groups in the total number of particles detected by scatter (p = 0.99); however, the number of E-stained fluorescent particles was significantly higher in ZIKV (p = 0.0007) and stock (p = 0.004) versus controls (Fig. 4G).

To further support the presence of ZIKV proteins as components of EVs released by infected cells, the ZIKV protein sequence was assessed by mass spectroscopy. Approximately 16% coverage of the ZIKV polyprotein was detected among the EV samples. A total of 69 unique peptides were detected (Supplementary CSV 1), which mainly align with ZIKV E (50 peptides) and nonstructural protein 1 (NS1; 14 peptides). Three peptides aligned with the capsid and membrane ZIKV structural proteins (Supplementary Fig. 6). In addition, one peptide aligned with NS4B and another peptide aligned to NS5. NS1, NS4B, and NS5 are not packaged into mature virions, which supports that ZIKV proteins are incorporated into trophoblast-secreted EVs. Minimal detection of the membrane or capsid proteins further supports minor Zika virion contamination as these proteins are highly abundant in Zika virions. Significantly more ZIKV peptides were detected in the TSC, ST3D, and EVT ZIKV-EV vs control EV samples (p = 6E−17, p = 1E−13, and p = 2E−6, respectively) (Fig. 4H). It should be noted that low quantities of sporadic ZIKV peptides were detected in control samples, possibly due to sample handling or background signal issues. Despite minimal detection in control samples, ZIKV peptides were significantly more abundant in ZIKV-EV samples. Altogether, these findings support the association of ZIKV proteins with trophoblast EVs.

**Figure 5:**
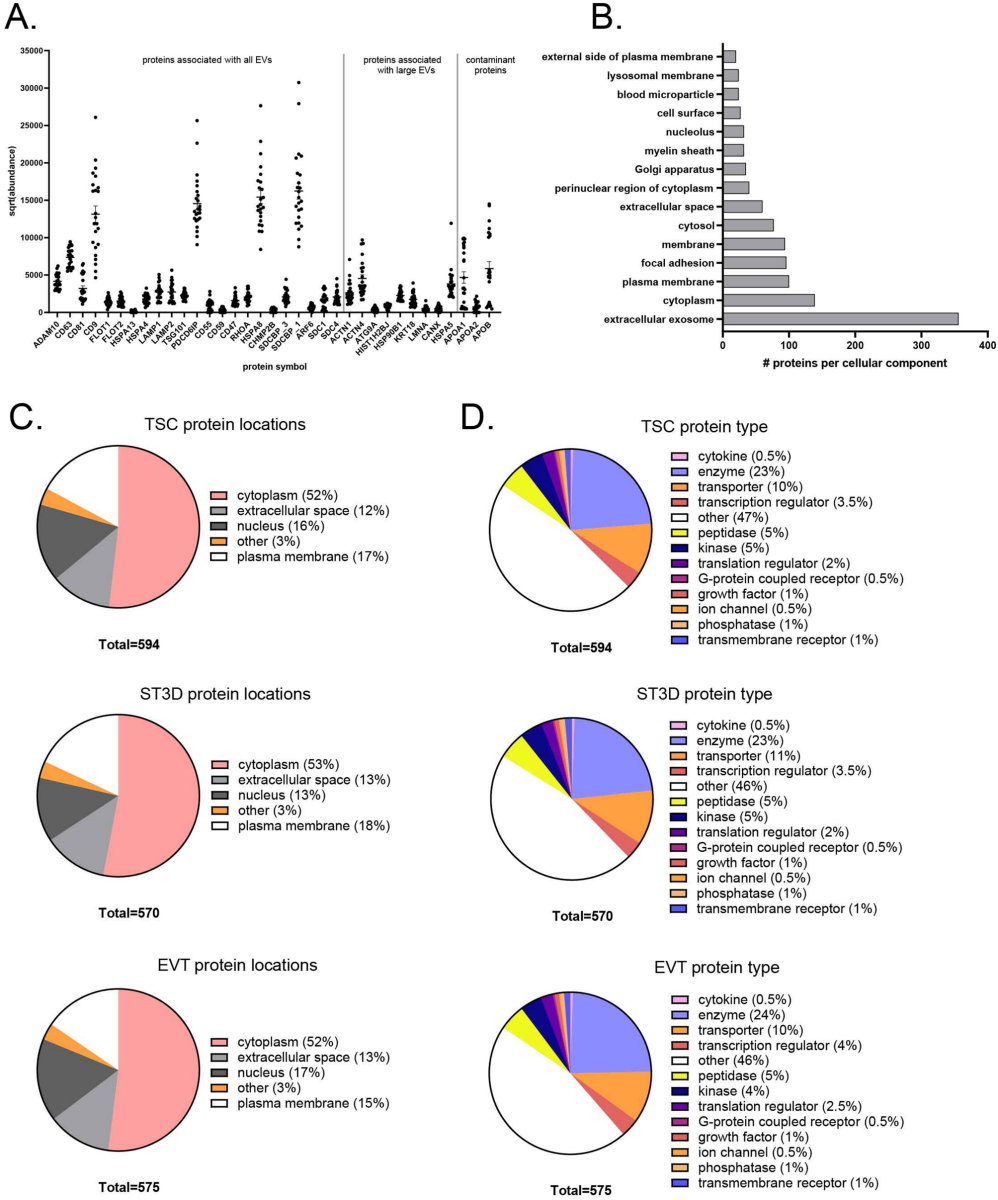
Characterization of protein cargo in trophoblast EV preparations. (**A**) The abundance of proteins associated with EVs, large EVs, and common contaminants is depicted. (**B**) DAVID pathways enrichment on the 763 proteins identified in all 24 EV samples; the top 15 components with an adjusted p-value of < 0.05 (Benjamini–Hochberg correction) are shown. (**C**,**D**) Protein location and type for all proteins detected in TSC, ST3D and EVT EVs as determined by IPA.

Additional analyses were completed to assess the degree of enrichment of EVs. Based on the MISEV2018 guidelines^42^, proteins commonly enriched in EV samples and common non-EV protein contaminants were identified in the mass spectroscopy data (Fig. 5A). CD9, PDCD6IP, HSPA8, and SDCBP isoform 1, proteins characteristic of all EVs, were the most abundant. The presence of proteins commonly seen in large EVs was also noted, suggesting that a mixture of EV types was present. Several apolipoproteins (APOA1, APOA2, and APOB) were detected, suggesting a low level of non-EV protein contamination. A total of 763 proteins were identified in all 24 EV samples (ZIKV and control) and were analyzed with DAVID enrichment analysis for additional characterization. The results confirmed high enrichment of the extracellular exosome cellular component (Fig. 5B). Other highly enriched cellular components included cytoplasm, focal adhesion, as well as membrane and plasma membrane, as expected. Lastly, IPA on all proteins detected in the TSC, ST3D, or EVT EV mass spectroscopy data showed across samples that ~ 50% of proteins isolated were cytoplasmic proteins and ~ 15–18% were plasma membrane-associated (Fig. 5C). Enzymes, transporter proteins, kinases, transcription regulators, translation regulators, and peptidases were some of the more abundantly detected protein types (Fig. 5D). Altogether, these data support the authenticity of the trophoblast-secreted EV preparations.

### Differential protein cargo in EVs secreted by ZIKV-exposed cells

**Figure 6:**
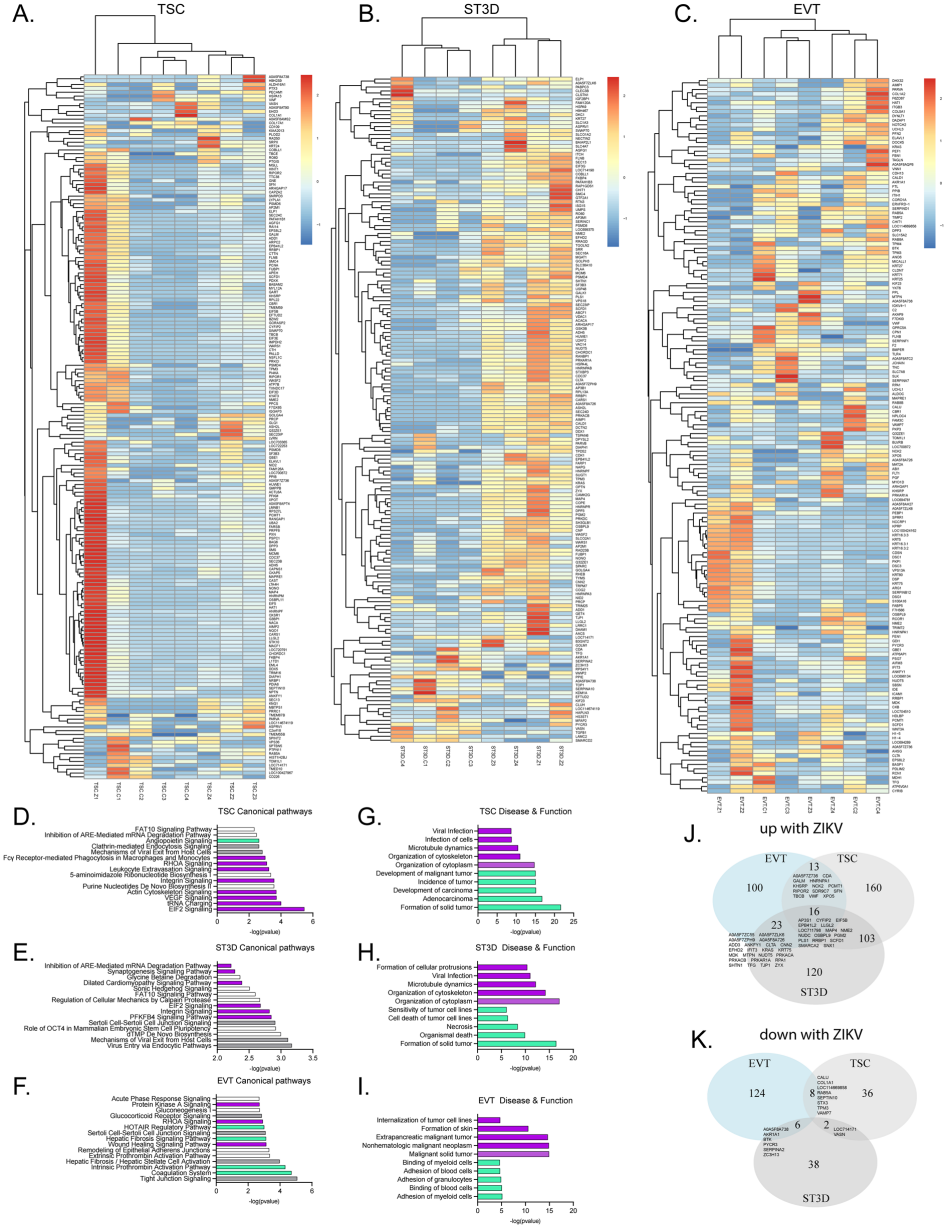
EV proteomics analysis. (**A**–**C**) Heatmaps of significantly differentially detected proteins present in at least half of TSC, ST3D, and EVT EV samples. (**D**–**F**) Top canonical pathways identified in the TSC, ST3D, and EVT EVs. Purple indicates a positive Z-score (activated); green indicates a negative Z-score (inhibited); white indicates 0 Z-score (no clear indication of whether the pathway is decreased or increased in ZIKV); and gray indicates unknown direction. (**G**–**I**) Top five predicted decrease (green) and top five predicted increase (purple) diseases and functions. (**J**,**K**) Venn diagrams of EV proteins increased or decreased with ZIKV exposure.

A total of 6940 proteins were identified across all EV samples. Proteomic analysis revealed that many proteins were significantly differentially detected in EVs from ZIKV-exposed versus control cells: TSC (338 total: 292 up, 46 down), ST3D (308 total: 262 up, 46 down), and EVT (290 total: 152 up, 138 down) (Fig. 6A–C). Heatmaps were generated for significantly differentially detected proteins that were present in at least half of the samples for each cell type. Full heatmaps, of all significantly differentially detected proteins, are present in Supplementary Fig. 7. TSC samples were predominantly clustered by infection status, with TSC-Z1 clustering closer to the controls but remaining separate. The ST3D samples clustered based on infection status, whereas EV protein from EVTs tended to cluster by cell line.

IPA was performed to predict the canonical pathways and disease and biological functions in which the EV proteins may have a role. The topmost significantly predicted canonical pathways increased in TSC-Z EVs were EIF2 signaling, tRNA charging, and VEGF signaling (Fig. 6D). For ST3Ds, virus entry via endocytic pathways and mechanisms of viral exit from host cells were the topmost predicted pathways (Fig. 6E). Unfortunately, it is unclear whether these pathways were increased or decreased. Interestingly, IPA predicted the wound healing signaling pathway was increased in EVT-Z EVs (Fig. 6F). Disease and biological function IPA predicted an increase in viral infection for TSC and ST3D EVs (Fig. 6G,H). EVT-EV analysis showed a decrease in binding and adhesion to various cells, including myeloid cells, blood cells, and granulocytes (Fig. 6I).

In search of EV biomarkers of infection, proteins significantly differentially detected by mass spectroscopy across the cell types were compared (Fig. 6J,K). There were 16 proteins increased and none decreased by ZIKV exposure in all three cell types. Functions of these proteins include protein transport (AP3S1, PLS1, RRBP1, SCFD1 and SNX1), vesicle transport (SCFD1), exocytosis (LLGL2), apoptosis (CYFIP2), and negative regulation cells growth (SMARCA2).

In terms of placenta-specific protein detection in EVs, MAMU-AG4 (Uniprot ID: Q5TM80), a macaque-specific placental MHC class I molecule, as well as pappalysin 1 (Uniprot ID: F6ZUN7) were detected in all 24 samples. With regard to other placenta-specific proteins, endogenous retrovirus group FRD member 1 (Uniprot ID: A0A1D5R0I7), was detected in 17 samples, placental growth factor (Uniprot ID: F7HB08) was detected in 13 samples, and pregnancy specific beta-1-glycoprotein 7 (Uniprot ID: F6YVT1) was detected in 6 of 8 EVT EV samples. Thus, these molecules could serve as markers of placenta-specific EVs in macaque trophoblast EVs.

### The impact of ZIKV on EV poly(A) cargo

A total of 24 EV samples were submitted for Poly(A)-seq but cDNA libraries could only be prepared for eight samples (3 TSC-C, 1 TSC-Z, 2 ST3D-C, and 2 ST3D-Z). Due to the limited data, samples were pooled into control or ZIKV. For the top 1000 genes identified in the eight EV poly(A)-seq samples, DAVID enrichment analysis showed that the most enriched cellular component was extracellular exosome (Supplementary Fig. 8A). A total of 31,459 transcripts were identified of which 2618 transcripts were detected in all eight EV samples. Conversely, 3898 and 9490 transcripts were specific to either ZIKV or control EV samples, respectively. When comparison was restricted to transcripts detected in a majority of samples regardless of cell type origin (3/5 control or 2/3 ZIKV-exposed; Supplementary Fig. 8B), 832 and 601 transcripts were detected only in control or ZIKV EVs, respectively. A total of 69 and 41 transcripts were identified in all three ZIKV EV samples or all five control EV samples, respectively. Immune response related genes were detected in EVs released by ZIKV-infected cells. IFNL1 transcript XM_015123826.2 was only detected in EVs released by ZIKV-infected cells. Two different IL1RN isoforms were detected in the control or ZIKV EVs, with each transcript being unique to either group (XM_001091833.4 and XM_015113137.2 in control or ZIKV, respectively). Pathways enriched by transcripts identified in the majority of ZIKV or control EVs (602 and 871 for ZIKV and control, respectively) are shown in Supplementary Fig. 8C. IPA analysis identified several pathways to be significantly increased (cholecystokinin/Gastrin-mediated, protein kinase A, senescence, and Rho family GTPase signaling) and decreased (RHOA, ILK, and PFKFB4 signaling; Supplementary Fig. 8C) by ZIKV exposure.

### The impact of ZIKV on EV miRNA cargo

**Figure 7:**
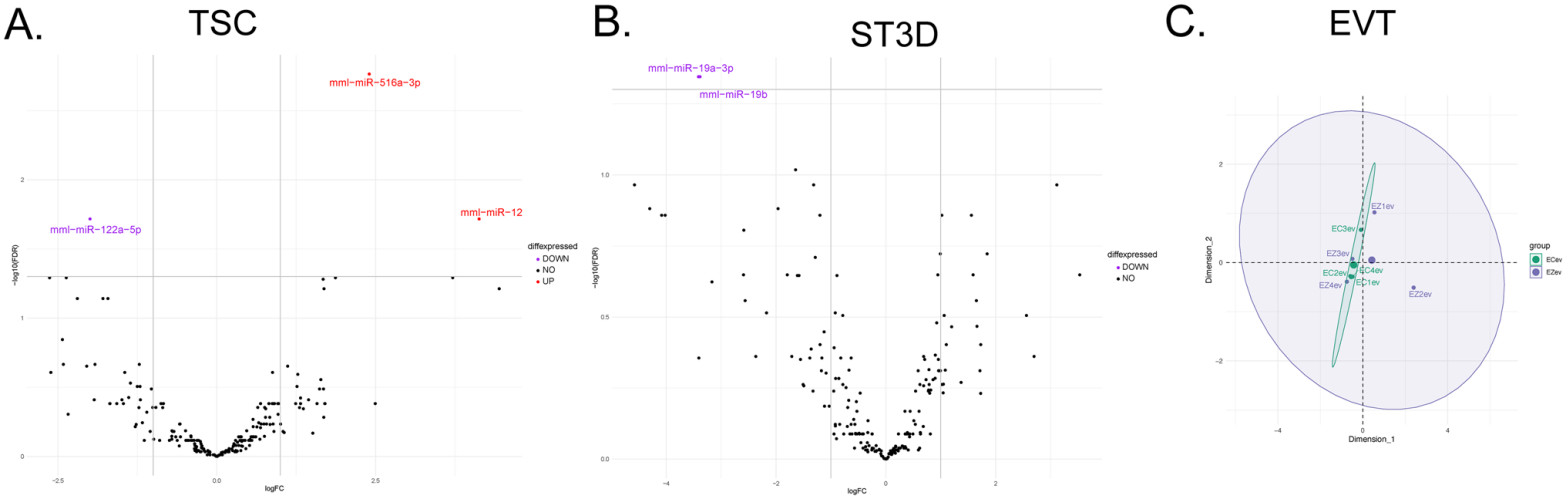
ZIKV exposure impact on EV miRNA cargo. Volcano plots of miRNAs detected in (**A**) TSC, and (**B**) ST3D EVs. miRNAs significantly differentially expressed are highlighted on the plots (Purple indicates decreased with ZIKV exposure; Red indicates increased with ZIKV exposure; Black indicates not significantly different). (**C**) An MDS plot of the EVT miRNA profile of Control (Green) and ZIKV exposed (Purple) samples.

miRNAseq detected 216, 201, and 169 miRNAs in the EVs released by TSC, ST3D, and EVTs, respectively. There were two (miR-516a and miR-1249) and one (miR-122a-5p) miRNA significantly increased and decreased, respectively, by ZIKV exposure in the TSC EVs (Fig. 7A). In addition, only two miRNAs (miR-19a-3p and miR-19b) were significantly decreased in ST3D EVs (Fig. 7B). Despite the many changes in EVT cellular miRNA expression, no miRNAs were significantly differentially represented in EVT EVs. The MDS plot of EVT EV miRNAs shows high overlap among the samples (Fig. 7C).

## Discussion

To determine which trophoblast cells are permissive to ZIKV infection and to understand how infection impacts function, we established a novel in vitro primate trophoblast cell model. TSCs and ST3Ds were highly permissive to infection as indicated by the presence of infectious virus in the culture medium and ZIKV proteins detected within cells. In addition, TSC and ST3D transcriptome enrichment analysis with the IPA “Disease and Function” tool indicated that these cells responded to ZIKV exposure. Conversely, EVTs maintained a level of resistance to ZIKV infection as evident by low virus production. ZIKV exposure also impacted EV size and cargo, and Zika viral proteins were detected in EVs by NTA and mass spectroscopy. The model used here supports the hypothesis that early gestation trophoblasts are permissive to ZIKV infection and that the impact of placental cell infection is reflected in the composition of trophoblast EVs. These results are consistent with reports of trophoblast permissiveness to ZIKV infection in human first trimester trophoblast cells, first trimester placental explants, human embryonic stem cell (ESC)-derived trophoblast cells, and human iPSCs^18,19,38,43^. Use of the macaque trophoblast model complements other nonhuman primate studies on infection of IVF-derived embryos^44^ and early pregnancy in vivo maternal infection^45,46^.

Cells maintain multiple mechanisms to identify and curtail a viral infection, including inhibiting protein translation, degrading viral mRNA transcripts, and alerting immune cells to infection via proinflammatory cytokine secretion^47,48^. ZIKV exposure induced expression of genes involved in a cellular immune response. For example, ISG20 expression was significantly increased in TSC-Z and ST3D-Z cells by poly(A)seq, and another report showed increased ISG20 in human first trimester trophoblast cells exposed to ZIKV^49^. The protective effect of IFNs against ZIKV infection also has been previously reported^50–52^. Elevated gene expression of IFNs and IFN pathway-associated genes was noted in the current study and agrees with previous studies^38,52–54^. While human ESCs-derived trophoblasts, which are described as primitive trophoblasts, expressed low levels of type I and III IFNs^19^, in the current study IFN-beta (type I IFN) and IFNL1 and IFL3 (type III IFNs) transcript abundance increased after ZIKV exposure. Provocatively, IFNL1 mRNA was detected in EVs from ZIKV-exposed but not control cells. The different responses between human ESC-derived trophoblasts and the rhesus TSC-derived trophoblasts in the current study suggests these two cell types represent different developmental stages. It should be noted that no changes in IFN concentrations measured by Luminex assay were detected in response to ZIKV infection and is potentially due to either post-transcriptional mRNA degradation or suboptimal cross-reactivity or sensitivity of the Luminex assay with the macaque proteins.

miRNAs also are important for trophoblast antiviral responses^55^. Unexpectedly, while the TSCs and ST3Ds were susceptible to infection, there were minimal changes in miRNA expression in these cells. One interpretation of these data is that that the cellular regulatory pathways that impact on miRNA expression are not affected by ZIKV in these cells. The fact that miRNA-seq identified expression of 325 miRNAs in TSCs, 333 miRNAs in ST3Ds, and 323 miRNAs in EVTs indicates that our analytical methods are indeed robust. Conversely, the predominant changes in miRNA expression were observed in EVTs, the cell type that appeared to control/limit ZIKV infection compared to TSCs or ST3Ds. This raises the possibility that these cells were able to respond more readily to viral exposure, and that changes in miRNA expression may be involved in controlling the infection. This possibility can be tested further with experiments in which TSC or STBs can be treated with miRNA mimics based on the miRNA profile of the EVTs.

Several miRNAs significantly increased in macaque EVTs by ZIKV exposure have also been reported in other models. miR-145-5p was significantly increased in the EVT-Z cell samples. miR-145-5p promotes cell proliferation, migration, and invasion in an EVT cell line^56^. In addition, miR-145-5p was upregulated by ZIKV infection in a neuroblastoma cell line and in brain tissue from still born fetuses with Congenital Zika Syndrome^57^. miRNA-195-5p was significantly increased in the EVT-Z cell samples and, based on the seed sequence, may impair Flaviviral infection as observed with similar miRNAs in HeLa cells^58^.

EVTs were able to maintain a level of resistance to ZIKV replication, which is unlike findings with other in vitro models^18,43,59^. The low level of EVT infection in the current study is an important finding as endovascular EVTs that migrate into and remodel maternal spiral arteries are in direct contact with maternal blood, circulating cells of the maternal immune system, and potentially ZIKV within maternal circulation. In addition they express the candidate ZIKV receptors along with two ligands potentially involved in ZIKV entry (GAS6 and PROS1)^19^. These findings suggest ZIKV could at least bind to these cells, an interpretation borne out by the change in EVT-Z EV cargo and the detection of ZIKV proteins in EVs by mass spectroscopy. One possible explanation for the minimal ZIKV replication in the EVT cultures may be the presence of extracellular matrix components (collagen IV and Matrigel). Experimental infection of cancer cell lines with herpes simplex virus type 1 in the presence of Matrigel resulted in decreased viral replication, and the authors concluded that the extracellular matrix hindered viral replication post-viral entry^60^. The point at which EVTs can control infection remains unclear and future studies are needed to fully elucidate their response to ZIKV exposure. Interestingly IPA predicted decreased adhesion and binding of various blood and immune cells from the EVT EV protein data. As mentioned previously, EVTs are exposed to the maternal blood and therefore changes in immune cell interaction could have downstream implications. Future study into the impact on EV protein cargo is of interest as these changes may impact the maternal immune response.

IPA analysis revealed several interesting similarities amongst the datasets. IPA of EV protein data from TSC and ST3D revealed activation of viral infection, microtubule dynamics, cytoskeleton organization, and cytoplasm organization as top canonical pathways increased by ZIKV. In addition, RHOA signaling was activated in the TSC-Z and EVT-Z EV protein data. The RHOA signaling pathway was reported to induce endothelial barrier dysfunction, increase vascular permeability, and increase the risk of Ebola virus disease^61^. Vascular damage is frequently observed in placentas impacted by ZIKV^45,62,63^. Further studies are needed to understand how EV signaling may impact endothelial barriers and vascular permeability. Interestingly, the EV mRNA cargo data suggest RHOA signaling was actually significantly inhibited in ZIKV EV samples. Due to the limited poly(A)seq EV data, further characterization of mRNA transcript presence in EVs is needed to better understand the implications of these inconsistencies.

Ultimately, the identification of biomarkers from circulating EVs could allow insight into placental health, or in the context of ZIKV, placental infection. Putative biomarkers identified in this study included the detection of ZIKV protein in EVs in addition to changes in EV cargo. Here, EVs within the EV size range stained positively for the ZIKV E protein by NTA, the ZIKV E protein was detected in EV samples by Western Blot, and several ZIKV proteins were detected in EV samples by mass spectroscopy. Although a low abundance of virions in the EV samples cannot be ruled out, the size of the ZIKV-E positive particles suggests incorporation of the E protein into EVs. In addition, the minimal detection of the ZIKV structural proteins, capsid and membrane, as well as the detection in EVs of NS proteins (NS1, NS4B, and NS5) that are not packaged in mature virions together support our conclusion of limited virion contamination in the EVs. The overlap between the ZIKV life cycle and exosome formation^41,64^ supports the potential for Zika viral proteins, genomes, and/or whole virions to be packaged within exosomes. Studies with other viruses, such as Dengue virus^24^, have demonstrated that viral proteins, genomes, and whole virions can be packaged into EVs.

Along these lines, viral infection was predicted to be increased in TSC-Z and ST3D-Z EV samples by IPA of the mass spectroscopy data. This finding supports the concept that a panel of biomolecules could be used to predict placental ZIKV infection. While the mechanism behind the packaging of these proteins into EVs remains unclear, it raises questions as to the significance of their presence. Future studies are needed to determine if these proteins were packaged to aid the infected cell via removal of proteins that enhance infection, or whether they were packaged and shipped out to increase the susceptibility of other uninfected cells, and therefore aid in viral dissemination. Studies with other viruses show that EVs can aid in both decreasing and increasing recipient cell susceptibility^23^.

Overall, this study shows that macaque trophoblasts representative of the early first trimester placenta are permissive to ZIKV infection. STs are in direct contact with maternal blood and therefore could be readily infected. While the location and presence of the TSC niche in the human or primate placenta is poorly understood^65^, these cells are not in direct contact with maternal blood but could become infected if the ST layer is breached, which is known to occur early in pregnancy^27^. As a progenitor cell that will differentiate to vCTB or column cytotrophoblasts to then give rise to ST and EVTs^66^, their permissiveness to ZIKV could have major implications on placental health. In addition, we saw that infection altered trophoblast function, even in EVTs that did not show prominent infection. ZIKV exposure also altered EV composition and EVs released by infected cells may contain ZIKV proteins. Altogether, these changes could impact the maternal response to the pregnancy and maternal immune response to ZIKV at the maternal–fetal interface. Furthermore, alterations in EV composition could be used to noninvasively identify placental ZIKV infection, which could be of significant clinical importance, not only for ZIKV but other TORCHZ infections.

## Methods

### Trophoblast cell culture

Rhesus monkey TSCs^12^ were graciously provided by Dr. Jenna Kropp Schmidt. The four TSC lines used for this study were: rh010319 (male; gestation date (gd) 58; line 1); rh090419 (female; gd 75; line 2), r121118 (male; gd 62; line 3), and rh020119 (female; gd 74; line 4). Previously published protocols^12^ were followed with some minor modifications as described. TSCs were differentiated into either EVTs or ST3D aggregates (Supplementary Fig. 1A). TSCs were grown in 13 ml media, EVTs in 15 ml media, and ST3Ds in 10 ml media (Supplementary Fig. 1B).

TSCs were differentiated to EVT by seeding into T75 flasks coated with 1 µg/ml collagen-IV (col IV; Corning, NY, USA, Cat #354233) with 2% Matrigel (Corning, Cat # 354234) added to the EVT media. No additional Matrigel was added on day 3 when the media were changed as previously described. Growth Factor Reduced Matrigel (0.5%; Corning, Cat #354230) was added on day 6.

For ST3D culture, TSCs were plated in non-adherent T75 flasks (Thermo Fisher, Cat #174952 and Cat #156800) and cultured for 5 days total. Three days after initial plating, the media were removed, and fresh media added. For additional details on the media composition and growth conditions, please see Schmidt et al.^12^.

### ZIKV stock

ZIKV, DAK AR 41524, NR-50338 (“DAKAR”) was obtained through BEI Resources, NIAID, NIH, as part of the WRCEVA program. To propagate this stock for subsequent experiments, the previously published protocol was followed^67^ with minor adaptations. Briefly, Vero cells (ATCC, Manassas, VA, USA, Cat #CCL-81) were plated at 4 × 10^6^ cells/T75 flask in MEM (ThermoFisher, Cat #11-095-080) containing 2% FBS (Peak Serum, Wellington, Colorado, USA, Cat #PS-FB1) and 100 mM sodium pyruvate (Sigma Aldrich, St. Louis, MO, USA, Cat #2256). The following day, they were exposed to DAKAR at an MOI of 0.1 for 1 h in 2 ml of MEM medium. Cytopathic effect was observed at 48 h in the ZIKV exposed cultures, and media were collected and spun at 15,000×*g* for 30 min at 4 °C. The supernatants were aliquoted and frozen at − 80 °C until used for infection studies. The final stock was sequenced by Dr. Shelby O’Connor’s laboratory at the University of Wisconsin-Madison using an Illumina MiSeq instrument and compared to the ZIKV stock reference sequence (Genbank Accession KY348860). Three single nucleotide (nt) substitutions at nt positions 470 (synonymous mutation; serine → serine), 3868 (missense, alanine–valine), and 3790 (missense, alanine–valine) were identified in this ZIKV stock. The frequencies of these substitutions were all below 16%. The stock concentration (4.6 × 10^7^ PFU/ml) was determined by plaque assays that were run in triplicate, as previously described^44^.

### Trophoblast ZIKV inoculations

Duration of culture and MOI were first optimized prior to generating experimental infection replicates (Fig. 1A). Both ZIKV-infected cells and uninfected controls underwent the same processing except uninfected cells were exposed to ZIKV-free Vero cell conditioned media that was collected alongside the ZIKV stock (mock infected). The MOI used was determined based on the quantity of virus calculated from the Vero cell plaque assay described above.

For infection of the TSCs and EVTs, one flask of cells from each line was lifted and counted just prior to infection. For TSCs and EVTs, media were aspirated, and 1 ml of inoculum was added to the flask. Cultures were incubated at 37 °C and rocked gently every 15 min for 1 h. The inoculum was removed, and the cells were washed once with PBS followed by addition of fresh medium. The number of ST3D “cells” (at this point they are aggregates) present was based on the number of TSCs added to the flask three days prior (Supplementary Fig. 1B). To infect the ST3Ds that were grown in suspension, aggregates/cells were pelleted by centrifugation at 500×*g* for 3 min, the supernatant was removed, and the cells were resuspended in 300 µl of inoculum. Aggregates were incubated in the ZIKV inoculum for 2 h at 37 °C and gently every 30 min. Since ST3Ds are grown in suspension, a larger volume of inoculum was used, and the length of inoculation was extended to account for this increase. Next, 2 ml of PBS was added, the cells were re-pelleted by centrifugation as above, and the supernatant was removed. A volume of 10 ml ST3D medium was added to the cells, and they were then transferred back into a T75 flask.

Once fresh media were added back for all cell types, a 500 µl aliquot was immediately removed to serve as a baseline of initial viral titer in the culture. The aliquot was spun at 500×*g* for 5 min to remove any cellular debris and a plaque assay was conducted. For EVTs, Growth Factor Reduced Matrigel (0.5%; Corning, Cat #354230) was supplemented to the cultures after this step. EVTs were cultured for a day longer than previously reported^12^ (72 h total) to extend the duration of ZIKV infection. An MOI of 5, 10, and 5 were used to infect the TSCs, ST3Ds, and EVTs, respectively. Inoculated and mock-inoculated control TSCs were cultured for 60 h, while ST3Ds and EVTs were cultured for 48 and 72 h, respectively.

### Sample collection: cells and media

At the end of the culture period, TSCs were lifted, EVTs were scraped off the flasks, and ST3Ds were pelleted prior to aliquoting cells for RNA, DNA, and protein isolation (Supplementary Fig. 1A). Conditioned media collected from all flasks were spun at 500×*g* for 5 min to remove dead cells and debris, pooled, and then aliquoted and frozen back at − 80 °C for EV isolation, hormone analysis, Luminex assay, or plaque assay.

### EV isolation

To isolate EVs, 20 ml of medium was placed onto a concentration column (Vivaspin 20; Sartorius, Swedesboro, NJ, USA, Cat # 1208L91) and spun for 60 min at 3000×*g* at room temperature (RT). For EVT EV samples, due to a high density of Matrigel in the conditioned media, the media were first filtered using a 0.22 μm filter (Millipore Sigma, Cat # SLGP033RS) and then spun for 60–90 min. The concentrated sample was passed through a size exclusion column (Izon, Medford, MA, USA, Cat # SP5, serial #1000788) according to the manufacturer’s protocol and the 1.5 ml flow through was collected. The sample was then concentrated using an Amicon concentration column (Millipore Sigma, Cat # UFC801024) for 45–60 min at 3000×*g* at RT. Eight replicates of 20 ml media volumes were processed for TSC and EVT EV isolation and then combined into one sample. Only six replicates of ST3D media were combined. This sample was quantified and characterized thrice by Zetaview Nanoparticle Tracking Analysis (NTA; methods described below). The sample was then divided into three aliquots, one each for RNAseq by freezing in Qiazol (Qiagen, Germantown, MD, USA, Cat #217004), mass spectrometry by freezing in PBS (FisherScientific, Cat #BP3991), and Western Blot by freezing in Pierce RIPA buffer (Fisher Scientific, Waltham, MA, USA, Cat #P18990) with 1 × HALT (Thermo Fisher, Cat #78440).

### EV sample quantification and characterization

EVs were quantified by Zetaview NTA (Particle Metrix, Meerbusch, Germany) using the following parameters: minimum brightness = 23, maximum size = 800, minimum size = 8, tracelength = 16, nm/class = 4, class/decade = 64, sensitivity = 80, frame rate = 30, and shutter = 100. Each sample was analyzed thrice, and the coefficient of variation (CV) was calculated. The CV within each sample set was less than < 6%. Paired *t* tests with Bonferroni correction were done to determine significance.

To determine if Zika virions were present in the EV preparations or whether the ZIKV envelope (E) protein co-localized with EVs released by infected cells, samples were incubated in AlexaFluor-488 labeled anti-ZIKV E antibody (1:10 dilution) for 2 h at RT in the dark prior to quantification with the Zetaview NTA. Samples were diluted at least 1:1000 according to the Zetaview NTA fluorescence protocol. As a positive control, 5.5 ml ZIKV stock was put through the EV isolation protocol, stained with the conjugated E antibody, and evaluated by NTA following the same protocol as described for the EV preparations. The fluorescent settings were minimum brightness = 20, maximum size = 800, minimum size = 5, tracelength = 11, nm/class = 4, class/decade = 64, sensitivity = 80, frame rate = 30, and shutter = 100. Statistical analysis was done with unpaired Mann–Whitney tests.

### Transmission electron microscopy

For transmission electron microscopy (TEM) imaging of EVs, 6 EV samples (2 per cell type, 1 ZIKV and 1 control each) were fixed in 2% paraformaldehyde (PFA; Fisher Scientific, Cat #50-980-487) and processed as cited^68^. Fixed samples were then placed on Formvar-carbon coated electron microscopy grids. After 20 min the grids were washed with PBS. The grids were then placed in 1% glutaraldehyde for 5 min and washed in distilled water 8 times. Grids were transferred to a uranyl-oxalate solution (pH 7) for 5 min and then a methyl cellulose-UA solution for 10 min on ice. Samples were dried and imaged.

### Detection of ZIKV via immunofluorescence

To evaluate cells exposed to ZIKV by immunocytochemistry, additional cells were infected and cultured as described above. For staining, TSCs were cultured for 60 h on col IV coated coverslips (5 µg/ml), rinsed in PBS, and fixed with 4% PFA for 10 min. Previous work showed that EVTs did not grow well on glass coverslips. Thus, after culture, EVTs were lifted, rinsed in PBS, fixed in 4% PFA, and cytospun at 1000×*g* for 1 min onto coverslips. ST3Ds were cultured for 48 h, then transferred to wells containing col IV-coated coverslips (5 µg/ml) and allowed to attach for 2 h. The cells were rinsed with PBS and fixed in 4% PFA for 10 min. After fixation, all coverslipped cells were rinsed twice with PBS and stored at 4 °C in PBS. For immunostaining, the cells were permeabilized for 10 min with 0.1% Triton-100 (Millipore Sigma, cat #T-9284) and then blocked for 10 min with Background Punisher (Biocare Medical, Pacheco, CA, USA, Cat #BP974H). For antibody details please see Supplementary Table 1. Cells were incubated with either primary specific or rabbit IgG isotype control antibody diluted in DaVinci Green Diluent (Biomedical Care, PD900M) for 1 h at RT, washed 3 times at 5 min each with 0.1% Tween Tris-buffered saline (TBST), and then exposed to secondary rabbit antibody for 45 min at RT. Finally, the cells were stained with DAPI for 5 min, washed in Milli-Q water, and then coverslips were adhered to slides using ProLong Diamond Mountant (Fisher Scientific, Cat # P36961). The following day the sides were imaged using a Nikon confocal microscope and Elements software (Nikon, Tokyo, Japan).

### Lactate dehydrogenase (LDH) apoptosis assay

To determine if ZIKV induced cell death, an LDH (Cytotox96 non-radioactive cytotoxicity assay, Promega, Madison, WI, Cat #G1780) time course was completed on each cell type. For this assay, additional cells were inoculated at the same MOI and length of duration as previously stated and shown in Supplementary Fig. 1A.

TSCs (100,000 cells/well) were seeded into 24-well col IV coated plates (Corning, Cat #3527). The following day they were exposed to ZIKV or mock infected. EVTs were plated (100,000 cells/well) in collagen IV coated 24-well plates and differentiated in the wells following the previously stated media changes. ST3Ds were differentiated in non-adherent T25 flasks, and after they were exposed to ZIKV (day 3 of differentiation, as previously stated) they were transferred (three-quarters of a T25 flask/well) to non-adherent 24-well plates (Eppendorf, Hamburg, Germany, Cat #0030 722.019). Vero cells were plated in 24-well plates and then exposed to ZIKV at an MOI of 1, 5, or 10 for 1 h the following day. Cells were plated such that two to three ZIKV-exposed and two to three control wells were quantified at each time point.

After the ZIKV exposure (as previously determined for each cell type), 50 μl of media was placed into a 96-well plate to quantify released LDH. A volume of 50 µl of the CytoTox 96 reagent was added and the plate incubated for 30 min at RT in the dark. Finally, 50 µl of Stop Solution was added, and the plate was immediately read at 490 nm. The average among the two–three wells was calculated and the change in LDH in infected cells compared to controls was determined. To determine significance, LDH absorbance was averaged over time. Control and ZIKV absorbances were averaged over time and ZIKV and control were compared via paired *t* test for the TSC, ST3D, and EVT data.

### Plaque assay

To determine the quantity of virus in the conditioned media, plaque assays on Vero cells were conducted as previously reported^44^. Samples were assessed in duplicate (EVT) or triplicate (TSC and ST3D). Paired *t* test with Bonferroni correction was used to determine significance.

### Hormone quantification

Monkey chorionic gonadotropin (mCG) and progesterone assays were performed as previously reported^44^. Samples were run in duplicate and unconditioned media were analyzed to determine background mCG and progesterone. Hormone quantities were normalized to cellular DNA. The lower limit of detection for the mCG and progesterone assays are 0.1 ng/ml and 10 pg/ml, respectively. A 2-way repeated measures ANOVA with Bonferroni correction was used to determine significance on log transformed hormone data, with cell types and infection status as factors. Repeated measure comes from each cell line measurement before and after exposure.

### Total RNA isolation from cells

Total RNA was isolated from cells using an RNeasy kit (Qiagen, Cat #74104) following kit recommendations with modifications. A volume of 700 µl of Qiazol was added to the cell pellet and then frozen at − 80 °C until RNA extraction. The protocol was followed with the same minor adaptations as with the miRNeasy kit. A 15 min DNAse treatment was performed on the column using RNase-Free DNase (Qiagen, Cat # 79254) prior to washing the column.

### Total RNA isolation from EVs

Each EV sample was diluted in 5 volumes Qiazol and incubated for 5 min at RT prior to freezing at − 80 °C until RNA extraction. The miRNeasy serum/plasma kit (Qiagen, Cat #217184) was used to isolate total RNA from EVs following the manufacturer’s protocol with minor adaptations. The Qiazol with Chloroform was overlaid onto phase maker tubes (ThermoFisher, Cat #A33248) and then spun at 4 °C at 16,000×*g* for 15 min. In addition, two RPE washes of 500 µl were applied prior to RNA elution. All samples were eluted in 30 µl RNAse free water and the initial elution was placed back on the column and reeluted to increase RNA concentration. Sample concentration and purity was determined with the NanoDrop One (ThermoFisher, Cat # ND-ONE-W).

### Poly(A)RNAseq

RNA integrity was checked with Agilent Technologies 2100 Bioanalyzer. Poly(A) tail-containing mRNAs were purified using oligo-(dT) magnetic beads with two rounds of purification. After purification, poly(A) RNA was fragmented using a divalent cation buffer in elevated temperature. The poly(A) RNA cDNA sequencing library was prepared following Illumina’s TruSeq-stranded-mRNA sample preparation protocol. Quality control analysis and quantification of the sequencing library were performed using Agilent Technologies 2100 Bioanalyzer High Sensitivity DNA Chip. Pair-end sequencing reads of 150 bp reads were generated on an Illumina NovaSeq 6000 sequencing system.

For cell poly(A)seq data, sequencing reads were filtered to remove adaptors and primer sequences and to remove sequences with a quality score lower than 20^69^. The cleaned sequencing reads were aligned to the reference genome (GCF_003339765.1_Mmul_10) using the HISAT2 package^70^. Multiple alignments with a maximum of two mismatches were allowed for each read sequence (up to 20 by default). Transcript abundance estimation and differential expression analysis of aligned reads of individual samples were assembled using StringTie^71^. Transcriptomes from all samples were then merged to reconstruct a comprehensive transcriptome using a proprietary Perl script designed by LC Sciences LLC (Houston, Texas, USA). Following transcriptome reconstruction, raw read counts were filtered, normalized, and differential expression determined with DESeq2^72^.

For EV poly(A)seq data, sequences were filtered and adaptors removed with Cutadapt^69^. Salmon^73^ was used to obtain transcripts estimates. The mapping rate for the eight sequenceable samples was between 3.5–12% for all except one, which had a mapping rate of 86%. Due to the low quality we chose to assess purely based on transcript presence or absence in control/ZIKV EVs. EVs released by control TSCs and ST3Ds (five total samples) were combined and compared to EV data obtained from ZIKV exposed TSCs and ST3Ds (three total samples). Due to limited sample size, differential expression analysis was not performed.

### miRNA-seq

The total RNA quality and quantity was assessed using a Bioanalyzer 2100 (Agilent, CA, USA). Approximately 0.4–1 µg of total RNA were used to prepare small RNA cDNA libraries utilizing the specified protocol for the TruSeq Small RNA Sample Prep Kit (Illumina, San Diego, USA). Single-read sequencing of 50 bp was performed on an Illumina Hiseq 2500 at LC Sciences LLC (Houston, Texas, USA). Read trimming and analysis were performed by the University of Wisconsin Biotechnology Center. miRNA abundance estimations were done using the miARma-Seq miRNA-Seq workflow. Accessions for known miRNAs from the Mmul_8.0.1 were obtained from miRbase v22 (October 2018). Data were filtered with the edgeR filterByExpr function and statistical analysis was performed with the edgeRglm function with a paired design.

### Validation of gene expression by qRT-PCR

A second RNA sample was extracted to generate technical replicates to validate gene expression changes by qRT-PCR that were initially identified by Poly(A)-sequencing analysis. cDNA was synthesized with a SuperScript III First-Strand Synthesis kit (ThermoFisher, Cat # 18080051) using 1 µg RNA per reaction. The manufacturer’s protocol was followed and the Oligo dT primer was used. qRT-PCR was performed with iQ SYBR Green Supermix (Bio Rad, Cat #1708882) following the manufacturer’s recommendation for primer concentration, master mix formulation and primer concentration. Beta-actin mRNA was amplified in parallel to serve as the reference gene and the primer sequences are listed in Supplementary Table 2. cDNA was diluted 1:10 and reactions were run in triplicate. The cycling protocol indicated with the kit was followed. Beta-actin was used to normalize and the 2^−ΔΔCt^ method^74^ was used to determine changes in transcript abundance*.* A 2-way repeated measures ANOVA with Bonferroni correction was used to determine significance, with cell types and infection status as factors. Repeated measure comes from each cell line measurement before and after exposure.

### Protein quantification and sample preparation

EV and cell protein samples for Western Blots were solubilized in Pierce RIPA buffer with 1 × HALT, sonicated for 3–6 s, spun down at 10,000×*g* for 10 min, and frozen at − 80 °C. Samples were quantified using the BCA assay as previously described^12^. For cell samples, 2–4 samples were quantified to estimate the total amount of cell protein in the study.

### Western blots

EV samples were diluted in 2 or 4 × TPA buffer (2 × recipe: 20 mM TRIS, 2 mM EDTA, 1 mM Na_3_VO_4_, 2 mM DTT, 2% SDS, 20% glycerol, and a few drops of bromophenol blue) and heated for 5 min at 70 °C. Cluster of differentiation (CD) 9, Heat shock protein 70 (Hsp70), and calnexin were stained for based on the MISEV 2018 guidelines^42^. To detect ZIKV, the E and nonstructural protein 2B (NS2B) antibodies were used; see Supplementary Table 1 for antibody information.

A total of 3 µg of cell, EV, or an in-house quality control (QC) protein sample was added per well of a 12% polyacrylamide gel along with 8 µl PageRuler Standard (Thermo Fisher, Cat #26616). Samples were prepared such that 2 identical blots were run at the same time. Gels were run in Running buffer (National Diagnostics, Cat # EC870). Protein transfer was done using the GENIE Electrophoretic Transfer (IdeaScientific, Minneapolis, MN, USA) and Transfer buffer (National Diagnostics, Cat # EC880) with 20% methanol (Fisher Scientific, Cat #A412P-4). Protein was transferred onto PVDF paper (Millipore Sigma, Cat # IVPH00010, Billerica, MA, USA) for 90 min at 0.5 amps with a Power Pac HC power supply (Bio Rad, Cat #1645052). The blots were blocked at 4 °C overnight in 5% nonfat dry milk (RPI, Cat # M17200, Mount Prospect, Illinois, USA) in 1 × TBST. Blots were incubated in primary antibody (at the concentrations specified in Supplementary Table 1) for 1 h on a rocker in 5% milk at 37 °C. The blots were then washed in 1 × TBST three times for 5 min each at RT and then exposed to either mouse or rabbit secondary antibody for 1 h on a rocker in 5% milk at 37 °C. The blots were washed again in TBST three times for 5 min each at RT and then exposed to Immobilon Crescendo HRP (Millipore Sigma, Cat # WBLUR0100) for 3 min at RT and imaged in a Bio-Rad ChemiDoc XRS + with ImageLab software.

### Mass spectrometry for proteomics

Digests were desalted using OMIX C18 SPE cartridges (Agilent) per manufacturer protocol and eluted in 20 µl of 50/50/0.1% ACN/H_2_O/TFA. Dried to completion in the speed-vac and finally reconstituted in 20 µl of 0.1% formic acid. Samples were analyzed on Orbitrap Fusion™ Lumos™ Tribrid™ platform, where 1 µl was injected using Dionex UltiMate™3000 RSLCnano delivery system (ThermoFisher Scientific) equipped with an EASY-Spray™ electrospray source (held at constant 50 °C). Chromatography of peptides prior to mass spectral analysis was accomplished using capillary emitter columns (PepMap^®^ C18, 2 µM, 100 Å, 500 × 0.075 mm, Thermo Fisher Scientific). NanoHPLC system delivered solvents A: 0.1% (v/v) formic acid , and B: 80% (v/v) acetonitrile, 0.1% (v/v) formic acid at 0.30 µL/min to load the peptides at 2% (v/v) B, followed by quick 1 min gradient to 5% (v/v) B and gradual analytical gradient from 5% (v/v) B to 37.5% (v/v) B over 73 min when it concluded with rapid ramp to 95% (v/v) B for a 5 min flash-out. As peptides eluted from the HPLC-column/electrospray source survey MS scans were acquired in the Orbitrap with a resolution of 120,000 followed by HCD-type MS2 fragmentation into Ion Trap (32% collision energy) under ddMSnScan 1 s cycle time mode with peptides detected in the MS1 scan from 350 to 1600 m/z; redundancy was limited by dynamic exclusion and MIPS filter mode ON.

Lumos acquired MS/MS data files were searched using Proteome Discoverer (ver. 2.2.0.388) Sequest HT search engine against Uniprot *Rhesus macaque* proteome database (UP000006718, 11/19/2020 download, 44,378 total entries). Static cysteine carbamidomethylation, and variable methionine oxidation plus asparagine and glutamine deamidation, 2 tryptic miss-cleavages and peptide mass tolerances set at 15 ppm with fragment mass at 0.6 Da were selected. For detection of ZIKV proteins, the Uniprot Zika virus (ZIKV) reference proteome polyprotein (UP000054557, 1/12/2022 download, 1 polyprotein entry) was used. Peptide and protein identifications were accepted under strict 1% FDR cut offs with high confidence XCorr threshold of 2.3 for z = 2 and 2.3 for z = 3 and at least 2 PSMs per protein identification. Strict principles of parsimony were applied for protein grouping. Chromatograms were aligned for feature mapping and area-based quantification using unique and razor peptides. Normalization was performed on total peptide amount and scaling on all averages. A paired *t* test (background based) was done to determine statistical significance.

### Luminex assay on conditioned media

To quantify secreted immunomodulatory proteins and growth factors, conditioned media were analyzed with the Protcarta 37-plex (ThermoFisher, Cat #: EPX370-40045-901) as previously described^44^ and a custom Bio-Rad 6-plex based on the Human Inflammation Panel 1 (BioRad, Cat# 171AL001M). For both assays, samples were run in duplicate on a Bioplex 200 instrument (BioRad, Cat #171000201). Data were analyzed with the Bioplex Manager Software. The lower level of quantification for each analyte is listed in the figure legend and is based on the manufacturer’s quantifications. A 2-way repeated measures ANOVA with Bonferroni correction was used to determine significance, with cell types and infection status as factors. Repeated measure comes from each cell line measurement before and after exposure. Analyte quantities were normalized to DNA. Some analytes were log transformed prior to statistical analysis.

### DNA preparation

Cell pellets were frozen at − 80 °C until DNA extraction. DNA was extracted using a Qiagen FlexiGene kit (Qiagen, Cat # 51206) according to the “Isolation of DNA from cultured cells” protocol. Two to four samples were extracted per sample type and DNA was dissolved in 100–500 µl of the kit’s FG3 buffer. If the sample was viscous after the heat incubation, it was sonicated for 3–6 s on ice. DNA was quantified using a Nanodrop One spectrophotometer.

### Quantification and statistical analysis

Data are represented as the mean ± standard error of the mean (SEM) of four biological replicates. Bar graphs and line graphs were generated using GraphPad Prism version 9.3.1 for Windows, GraphPad Software, San Diego, California USA, http://www.graphpad.com. Heatmaps, volcano plots, and Venn diagrams were produced in R using the pheatmap^75^, ggVennDiagram^76^, and ggplot^77^ functions, respectively. Statistical analysis was performed using GraphPad Prism 9.0 unless indicated otherwise. For the mass spectrometry, Poly(A)seq, and miRNAseq data, significance was determined with an adjusted p-value < 0.05 and 1 < log_2_ fold change < − 1. For heatmaps, the rows were organized by hierarchical clustering using agglomerative clustering with Ward's minimum variance method and the Euclidean distance metric. The miRNA PCA plots were made with Clustvis^78^.

## Supplementary Information

Supplementary Information 1.

Supplementary Information 2.

## Data Availability

Poly(A)-seq and miRNAseq data have been deposited at GEO (GSE185113 and GSE193195, respectively) and are publicly available as of the date of publication. The EV poly(A)-seq data was deposited and is available at GEO (GSE185291). The mass spectrometry proteomics data have been deposited to the ProteomeXchange Consortium via the PRIDE^79^ partner repository with the dataset identifier PXD033289 and 10.6019/PXD033289.
