# Supplementary material for: Zika virus impacts extracellular vesicle composition and cellular gene expression in macaque early gestation trophoblasts
Source: Sci Rep. 2022 May 5;12:7348. doi: 10.1038/s41598-022-11275-9 (PMC9072346; doi:10.1038/s41598-022-11275-9)
Supplement: Supplementary file 1 — Supplementary Information 1. [file 41598_2022_11275_MOESM1_ESM.docx]

**Supplementary Figures & Tables**

*
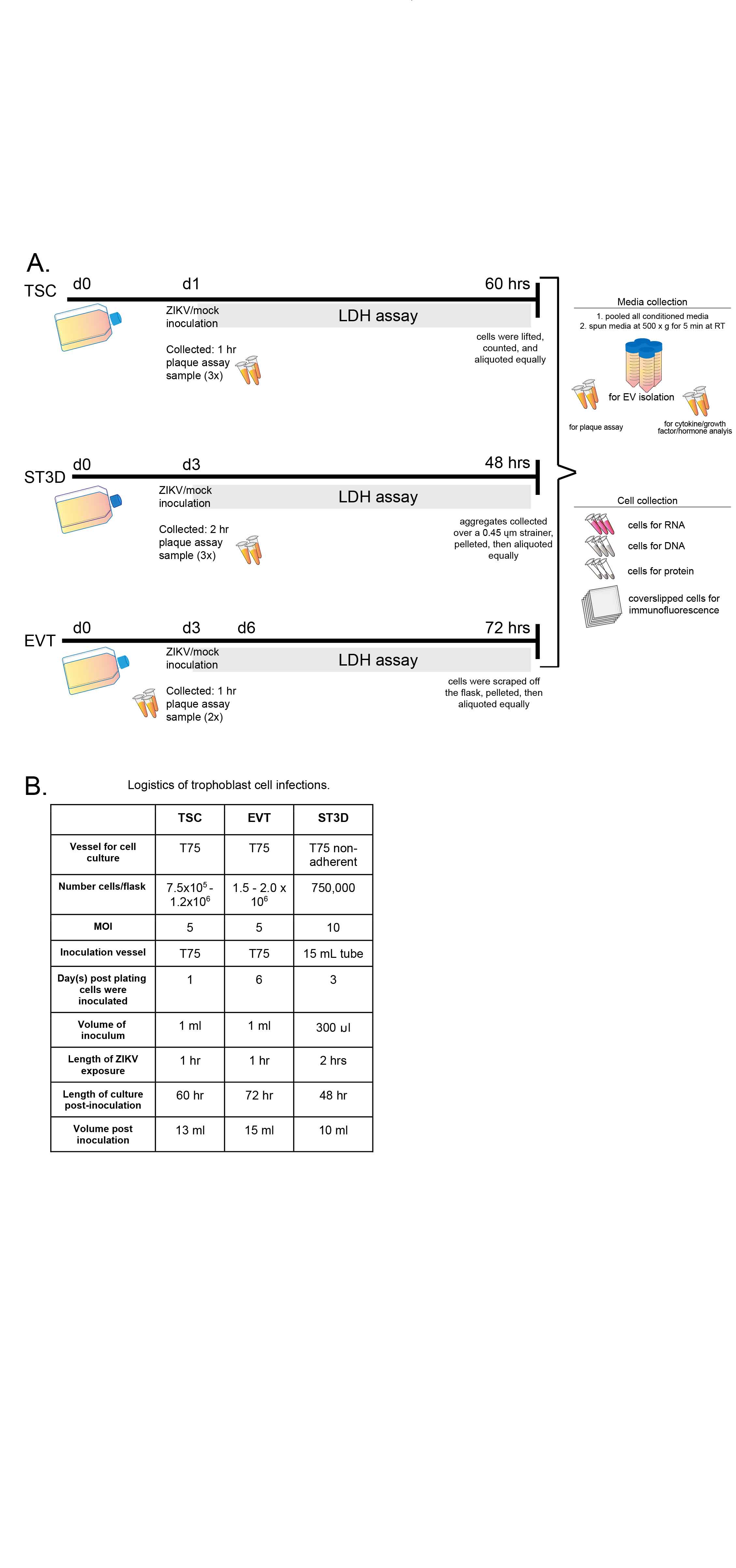
*

**Supplementary Figure 1. Methods overview. A)** A timeline depiction of how TSCs, ST3Ds, and EVTs were cultured, when samples were collected, and for what assay samples were collected. **B)** The logistics of trophoblast cell infection including the type of vessel used for cell culture, the number of cells plated, the MOI, inoculation vessel, the day of differentiation cells were inoculated, volume of inoculum, length of ZIKV exposure, length of culture post-inoculation, and media volume post inoculation.


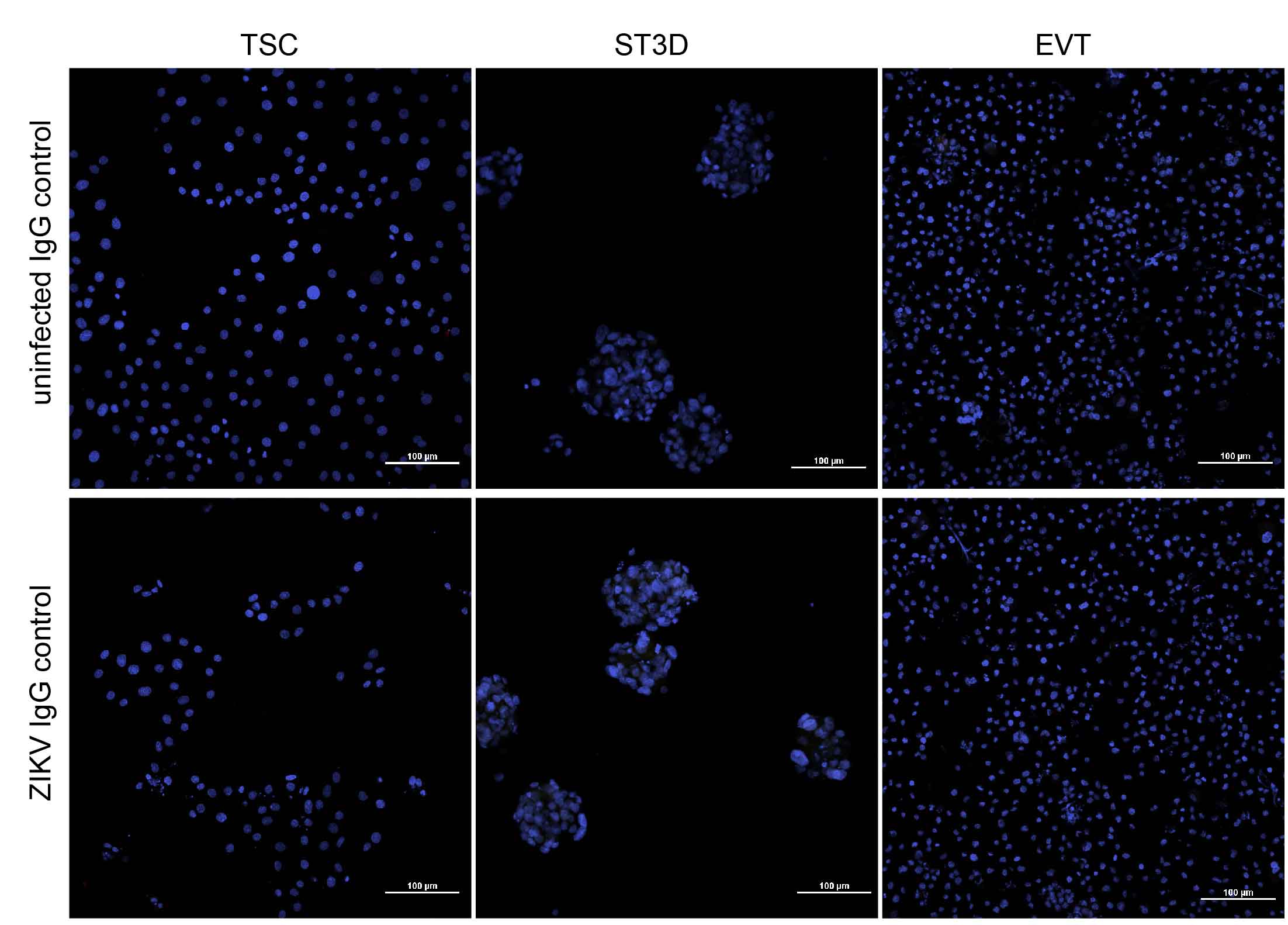


**Supplementary Figure 2. Immunocytochemistry images for rabbit IgG controls and ZIKV uninfected controls.** Rabbit IgG controls on TSC, ST3D, and EVT cells.


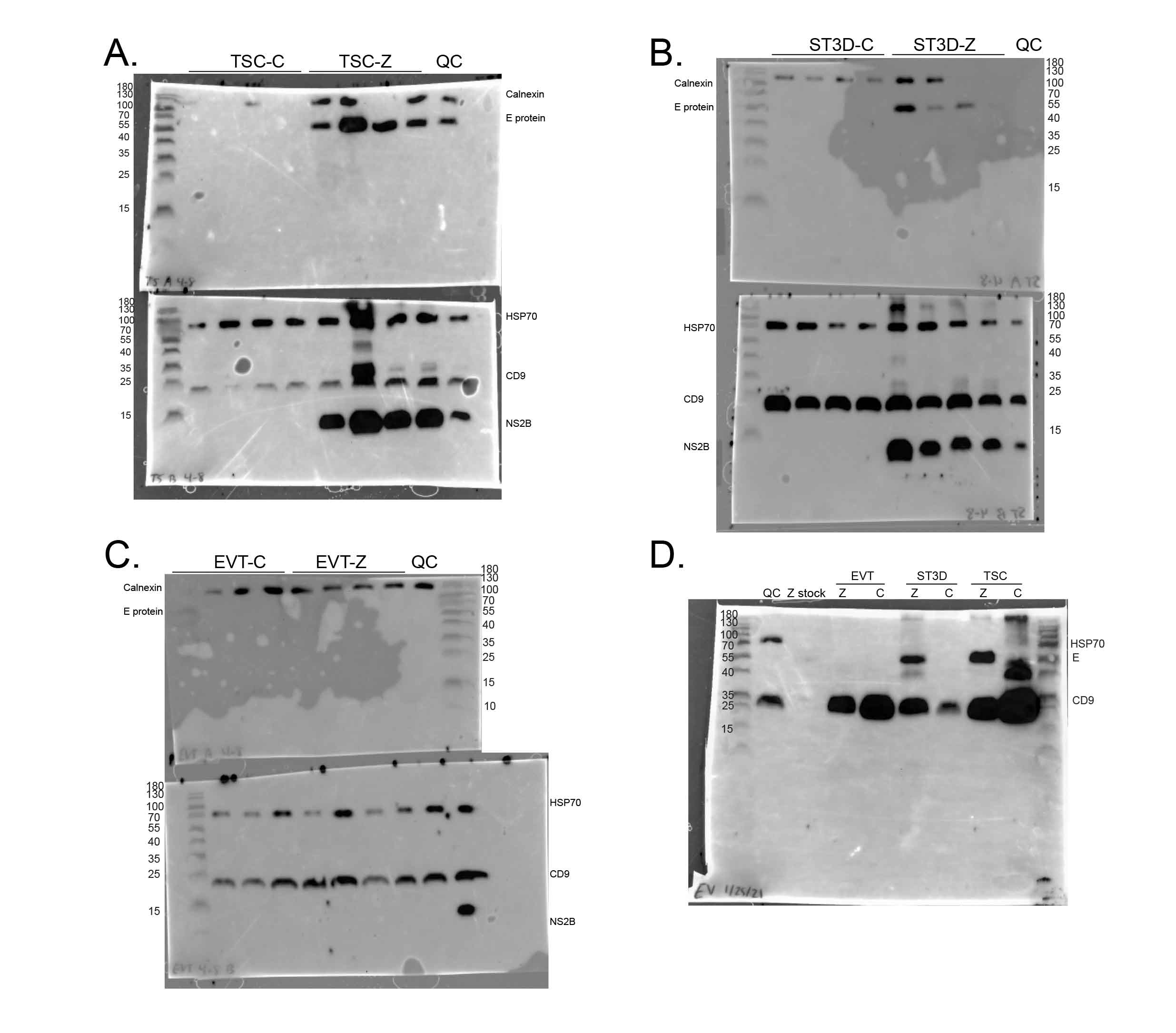


**Supplementary Figure 3.** **Full Western Blots. A-C)** The full Western Blots of TSCs, ST3Ds, EVTs lysates are shown with the ladder merged with the stained blot. **D)** The Western Blot completed on six of the EV lysate samples.


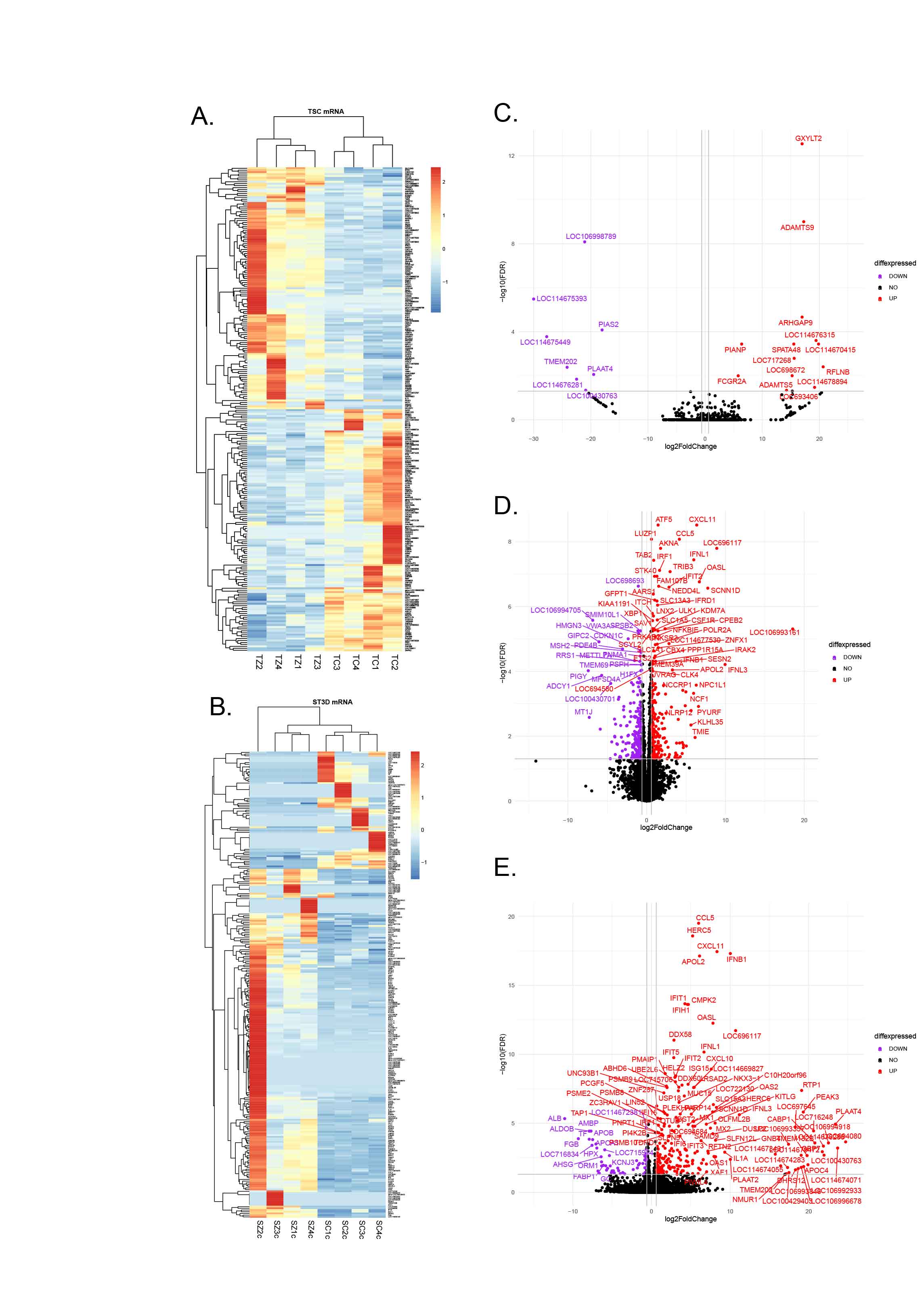


**Supplementary Figure 4. Cellular poly(A)seq analysis.** Heatmaps of all genes significantly differentially detected in **(A)** TSC and **(B)** ST3D samples. Volcano plots highlighting significantly upregulated (red) and downregulated (purple) genes for **(C)** EVT, **(D)** TSC, and **(E)** ST3D samples.


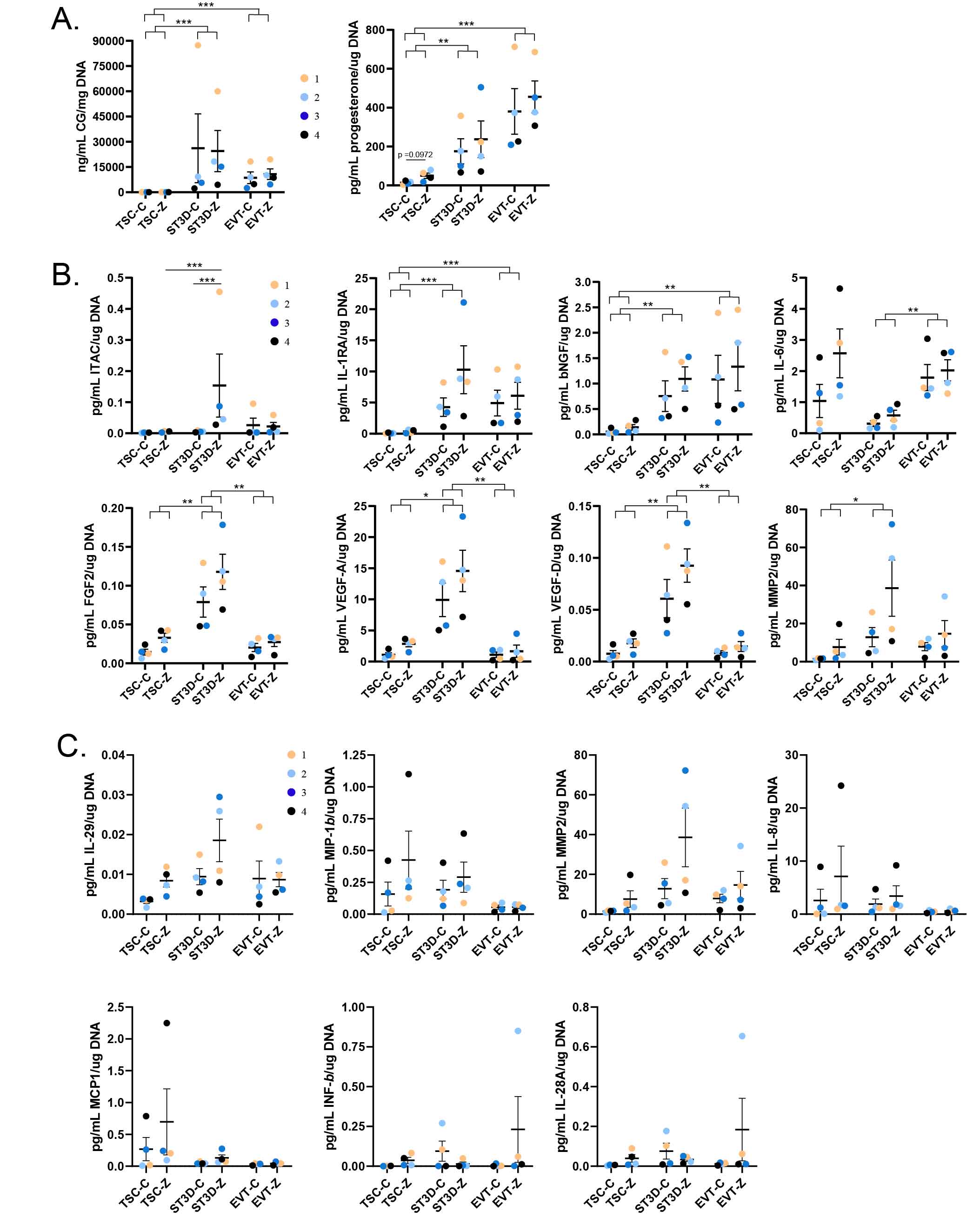


**Supplementary Figure 5. Hormone, cytokine, and growth factor secretion by cell type and infection. A)** Quantification of CG and progesterone secretion. **B)** Luminex assay data on analytes that were significantly impacted by ZIKV exposure (ITAC) or significantly different based on cell type. **C)** Luminex assay data on seven analytes (MIP-1b, MCP1, IFN-beta, MMP2, IL-8, IL-28A, and IL-29) which were detected in more than five samples but had no significant changes in expression. All secretions were normalized to cellular DNA quantity and, when necessary, data were log transformed prior to analysis. A 2-way repeated measures ANOVA with Bonferroni correction was used to determine significance, comparing cell types and infection status. Repeated measure comes from each cell line measurement before and after exposure. Analyte quantities were normalized to DNA content per well. Some analytes were log transformed prior to statistical analysis. Significance is depicted on the graphs (***** p <0.05; ** p <0.01; *** p <0.001).


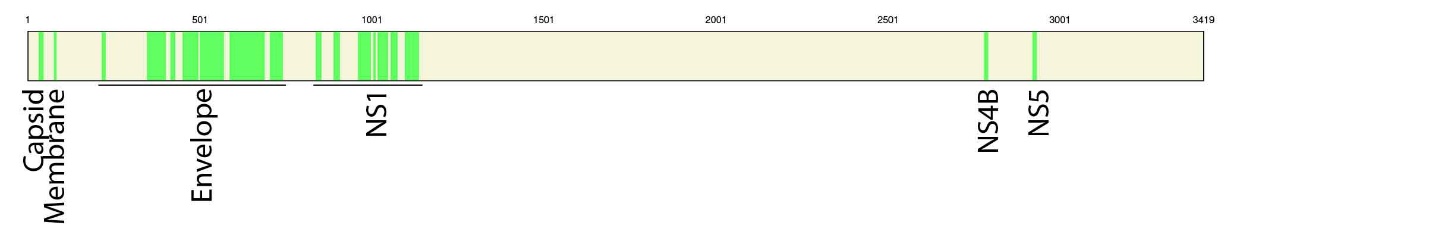


**Supplementary Figure 6. Alignment of ZIKV peptides to the polyprotein.** Peptides detected in EVs by mass spectroscopy are mapped to the ZIKV polyprotein in the Proteome Discoverer software. Peptides detected are highlighted in green, the width of the bar indicates the length of peptides detected. The numbers denote the amino acid position ^76^.

**
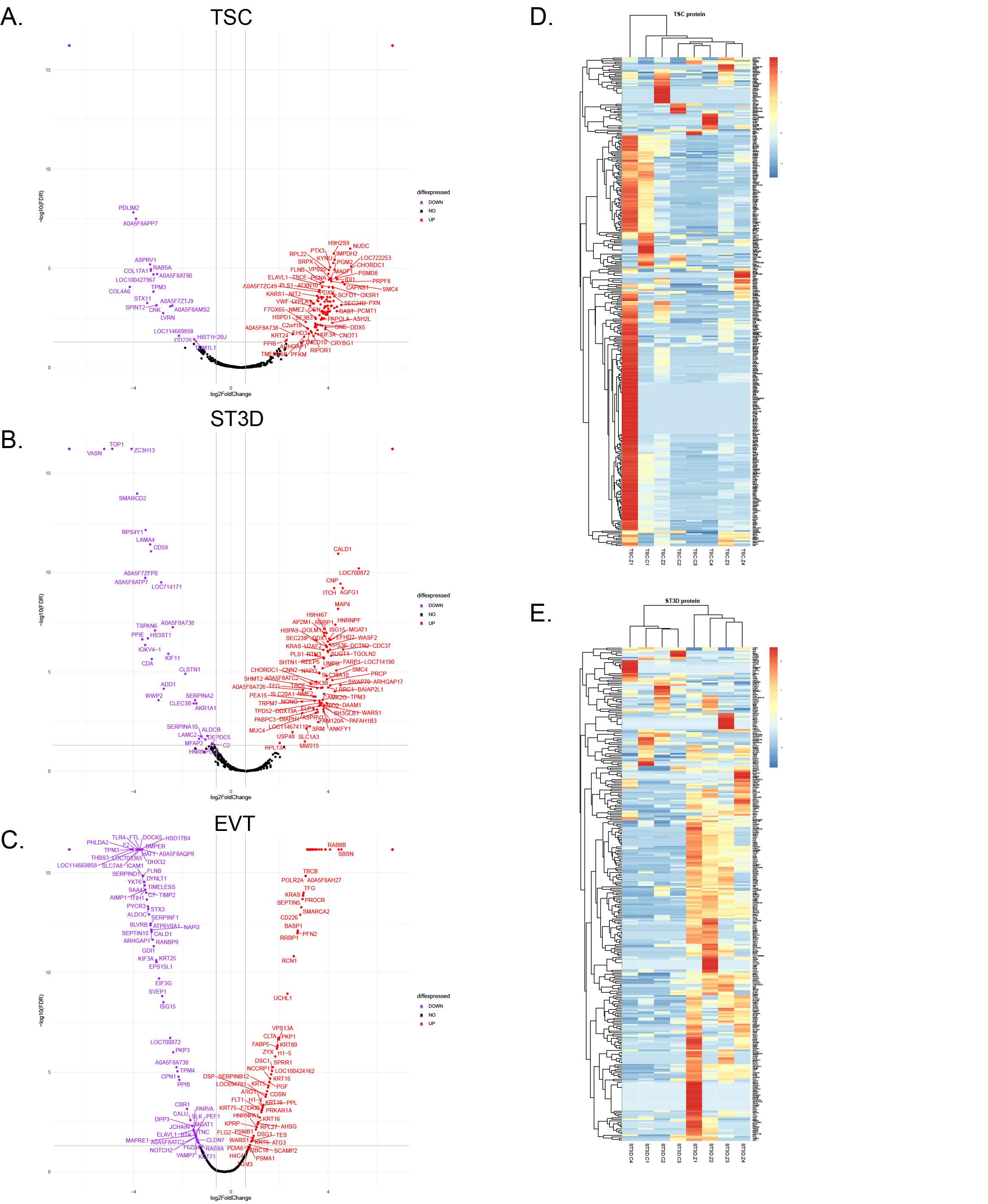
 Supplementary Figure 7.** **EV mass spectroscopy proteomics analysis.** Volcano plots highlighting significantly upregulated (red) and downregulated (purple) genes for **(A)** TSC, **(B)** ST3D, and **(C)** EVT samples. Heatmaps of all genes significantly differentially detected in **(D)** TSC and **(E)** ST3D samples.

**
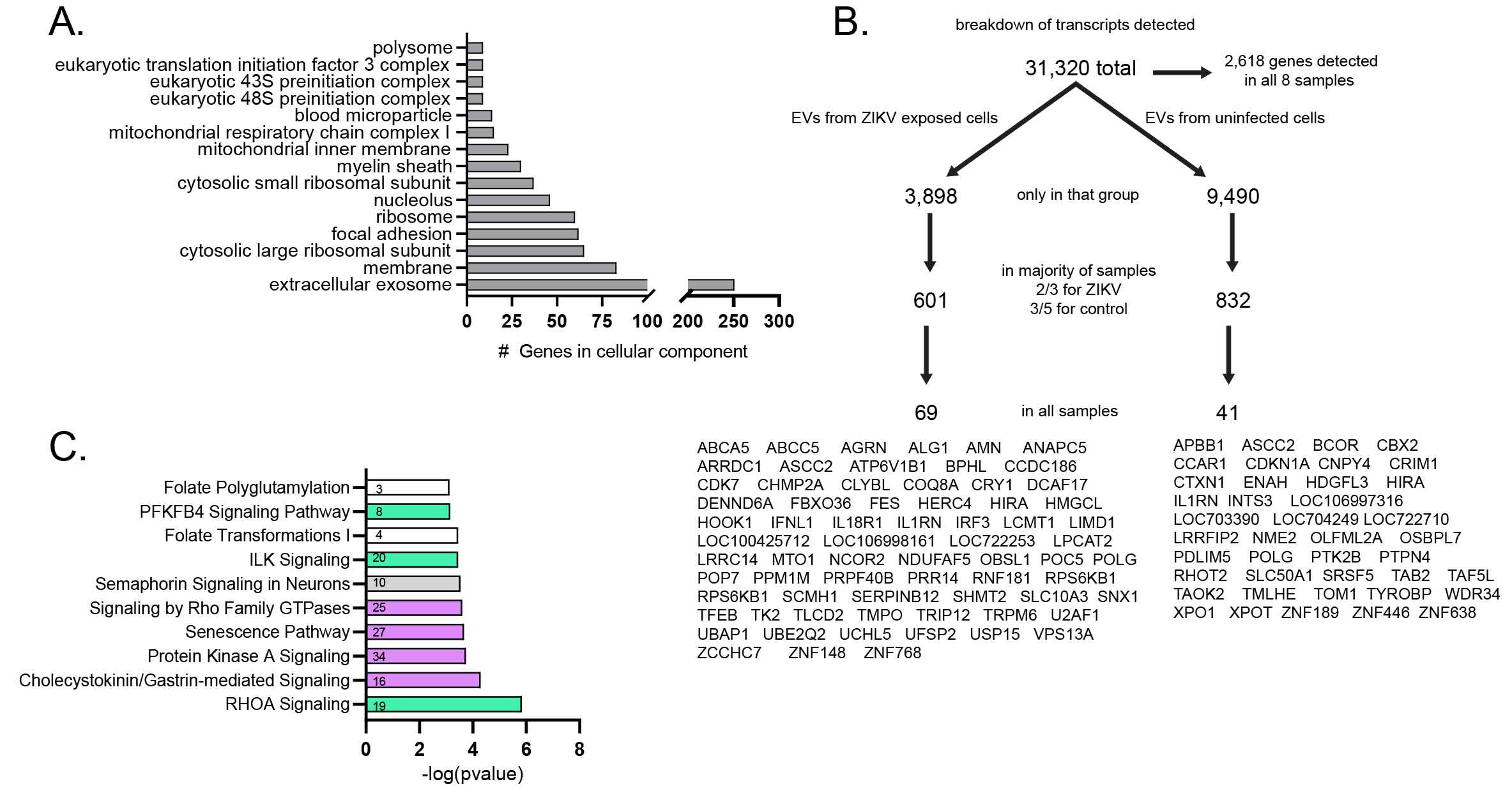
Supplementary Figure 8. ZIKV exposure impact on EV mRNA cargo.** **A)** DAVID enrichment analysis on the top 1000 genes detected in all eight samples. **B)** Top 10 canonical pathways identified in a majority of either the ZIKV or control EV samples (-log_10_ (p-value)). Purple indicates a positive Z-score; green indicates a negative Z-score; and white indicates 0 z-score (no clear indication of whether the pathway is decreased or increased in ZIKV). The number of transcripts identified in each pathway are stated on the graph. **C)** Schematic of the total number of transcripts detected across the eight EV samples, the number of transcripts solely detected in either the ZIKV or control samples, the number of transcripts detected in a majority (three of five control; two of three ZIKV samples) of the samples per group, and the number of transcripts detected in all samples of that group. The 66 and 35 gene IDs that correspond with transcripts identified are presented below.

**Supplementary Table 1**. Immunocytochemistry, fluorescent conjugates, and western blot antibodies. The purpose of the antibody, the target, purchasing information, and the concentration used are listed.

| **Antibody used for** | **Target** | **Vendor** | **Cat number** | **Clone** | **Stock Conc** | **Final conc** | **Lot number** | **Isotype** |
| --- | --- | --- | --- | --- | --- | --- | --- | --- |
| **IF Primary antibodies** | ZIKV envelope (4G2) | Novus Biological | NBP2-52666 | D1-4G2-4-15 | 1 mg/ml | 1:300 | T1902A20 | Rabbit IgG kappa |
|  | Rabbit IgG | Invitrogen | 31235 |  | 11mg/ml | 1:3300 | VG3028568 |  |
| **IF Secondary antibodies** | donkey anti-rabbit 647 | Jackson ImmunoResearch | 711-605-152 |  | 1mg/800ul | 1:833 | 127614 |  |
|  | donkey anti-rabbit 594 | Jackson ImmunoResearch | 711-585-152 |  | 1mg/800ul | 1:833 | 130064 |  |
| **Other** | Dapi diacetate | Invitrogen | D3571 |  | 10 mg/ml | 1 ug/ml | 1890543 |  |
| **Zetaview antibody** | ZIKV envelope | Novus Biological | NBP2-52709AFF488 | 4G2-4-15 | 0.68 mg/ml | 1:6.67 | T1908B05-020321-AF488 |  |
| **Western blot primary antibodies** | CD9 | Invitrogen | MA1-80307 | MM2/57 | 1 mg/ml | 1:2000 | UL2889567 & UC2744222 | Mouse |
|  | ZIKV E | GeneTex | 133314 |  | 0.45mg/ml | 1:900 | 43306 | Rabbit |
|  | Hsp70 | GeneTex | GTX111088 |  | 0.73 mg/ml | 1:730 | 43082 | Rabbit |
|  | NS2B | GeneTex | GTX133308 |  | 1.15 mg/ml | 1:1150 | 42564 | Rabbit |
|  | Calnexin-CT | StressMarq Biosciences | SPC-108B |  | 1 mg/ml | 1:1000 | PC185088 | Rabbit |
| **Western blot secondary antibodies** | anti-mouse | Jackson ImmunoResearch | 115-035-146 |  | -------- | 1:20,000 | 151651 |  |
|  | anti-rabbit | Jackson ImmunoResearch | 111-035-144 |  | -------- | 1:20,000 | 151080 |  |

**Supplementary Table 2.** Primer sequences for qRT-PCR. The forward and reverse primer sequences for each gene quantified are listed as well as the amplicon size.

| Gene | Forward (5’ -> 3’) | Reverse (5’ -> 3’) | Amplicon size |
| --- | --- | --- | --- |
| ACTB | CTA CCA TGA GCT GCG TGT GG | GTA CAT GGC TGG GGT GTT GA | 130 |
| INFL1 | ATC GTG GTG CTG GTG ACT TT | TTG AGT GAC TCT TCC AAG GCA | 170 |
| ISG20 | TGC TGT GCT GTA CGA CAA GT | CCA AGC AGG CTG TTC TGG AT | 339 |
| OASL | CAT CGT GCC TGC CTA CAG AG | GAC CTG GCT TTC ACA TAC TGC T | 219 |
| DDX58 | CTG GTT CCG TGG CTT TTT GG | CCC CTT AGT GGA GCA AAT CTG T | 238 |
| IFNB1 | CTC CTG TTG TGC TTC TCC ACT | AAT GCA GCG TCC TCC TTC TG | 203 |
| DHX58 | AAG ATC CTG CAA AGG CAG TTC A | TAC TTC TTG CTG GTC  CCT CTG G | 204 |
| PARP14 | CCA AGA ATG GCC AGA CAA TGA | CTT TCC ATA TGC CAC AGC ATT C | 128 |
| MT1E | CTG GCT GCG TTT TTG CCT TA | TCT TCT TGC AGG AGG TGC ATT | 147 |
| MTX1 | CGT GTT TGC CTC TTC ATT GGG | CAC AGG AAC CAT CAG GCG AG | 86 |

**Supplementary Table 3.** qRT-PCR and DESeq2 log fold change comparisons. The log2fold change for each gene listed is provided and split up based on cell type.

|  | TSC | | ST3D | | EVT | |
| --- | --- | --- | --- | --- | --- | --- |
| Gene name | qRT-PCR | DESeq2 | qRT-PCR | DESeq2 | qRT-PCR | DESeq2 |
| PARP14 | 2.8 | 2.25 | 3.9 | 3.65 | 1.5 | 1.05 |
| ISG20 | 5.9 | 4.00 | 5.3 | 4.44 | 0.4 | -0.04 |
| DHX58 | 2 | 1.79 | 3.8 | 2.88 | 1.6 | 1.09 |
| INFL1 | 9.1 | 5.98 | 9.4 | 6.67 | 2.3 | 1.12 |
| INFB1 | 6.7 | 3.75 | 9.9 | 10.02 | 2.3 | 3.18 |
| MT1E | -2.3 | -2.22 | 0 | 0.44 | 0.4 | 0.21 |
| MT1X | -1.8 | -3.30 | 0.5 | 0.89 | 0.8 | 0.99 |
| OASL | 5.8 | 6.68 | 8 | 7.82 | 2 | 1.97 |
| DDX58 | 1.8 | 1.50 | 3.9 | 2.85 | 1.5 | 0.82 |

**Supplementary Table 4.** Significance (Bonferroni adjusted p-value) and Confidence Interval (C.I.) of mRNAs assessed by qRT-PCR. The p-value and C.I. of each mRNA assessed by qRT-PCR are listed below.

| mRNA by qRT-PCR / adj p-value (C.I.) | TSC-C vs TSC-Z | ST3D-C vs ST3D-Z | EVT-C vs EVT-Z | TSC-C vs ST3D-C | TSC-C vs EVT-C | ST3D-C vs EVT-C | TSC-Z vs ST3D-Z | TSC-Z vs EVT-Z | ST3D-Z vs EVT-Z |
| --- | --- | --- | --- | --- | --- | --- | --- | --- | --- |
| ISG20 | <0.001  (4.43 to 7.41) | <0.001  (3.81 to 6.79) | 1  (-1.13 to 1.85) | 0.060  (0.84 to 4.53) | 0.002  (2.32 to 6.01) | 0.985  (-0.36 to 3.32) | 0.270  (0.22 to 3.91) | 1.175  (-3.23 to 0.45) | 0.009  (-5.30 to -1.61) |
| DHX58 | 0.035  (0.84 to 3.24) | <0.001  (2.64 to 5.04) | 0.135  (0.39 to 2.79) | 1  (-1.98 to 2.10) | 0.327  (0.16 to 4.24) | 0.367  (0.10 to 4.18) | 0.647  (-0.18 to 3.90) | 0.796  (-0.29 to 3.80) | 1  (-2.15 to 1.94) |
| IFNL1 | <0.001  (7.57 to 10.71) | <0.001  (7.86 to 10.99) | 0.086  (0.71 to 3.84) | 1  (-2.03 to 3.48) | 0.113  (0.88 to 6.40) | 0.353  (0.16 to 5.67) | 1  (-1.75 to 3.76) | 0.218  (-5.98 to -0.47) | 0.042  (-6.99 to -1.48) |
| MT1E | 0.011  (-3.44 to -1.19) | 1  (-1.08 to 1.17) | 1  (-0.77 to 1.48) | 1  (-1.01 to 2.57) | 0.524  (-0.07 to 3.51) | 1  (-0.85 to 2.73) | 0.015  (1.35 to 4.93) | <0.001  (2.61 to 6.18) | 1  (-0.54 to 3.04) |
| MTX1 | 0.148  (-3.18 to -0.42) | 1  (-0.86 to 1.90) | 1  (-0.59 to 2.17) | 1  (-1.46 to 4.00) | 1  (-2.07 to 3.39) | 1  (-3.34 to 2.12) | 0.116  (0.86 to 6.31) | 0.202  (0.52 to 5.97) | 1  (-3.07 to 2.39) |
| DDX58 | 0.041  (0.70 to 2.83) | <0.001  (2.84 to 4.96) | 0.095  (0.45 to 2.58) | 1  (-1.00 to 1.68) | 0.169  (0.31 to 2.98) | 0.495  (-0.03 to 2.65) | 0.001  (1.13 to 3.81) | 0.383  (0.05 to 2.73) | 0.966  (-2.42 to 0.26) |
| IFNbeta1 | <0.001  (5.37 to 8.11) | <0.001  (8.57 to 11.31) | 0.042  (0.89 to 3.63) | 0.061  (-4.60 to -0.85) | 1  (-2.74 to 1.02) | 0.457  (-0.01 to 3.74) | 1  (-1.41 to 2.34) | <0.001  (-7.217 to -3.468) | <0.001  (-7.69 to -3.9) |
| OASL | <0.001  (3.97 to 7.65) | <0.001  (6.21 to 9.88) | 0.304  (0.19 to 3.87) | 1  (-1.38 to 4.28) | 0.168  (0.65 to 6.32) | 1  (-0.80 to 4.87) | 0.122  (0.86 to 6.52) | 1  (-3.12 to 2.54) | 0.077  (-6.81 to -1.15) |

| mRNA by qRT-PCR/ adj p-value (C.I.) | TS vs ST3D | TS vs EVT | ST3D vs EVT | Control vs ZIKV |
| --- | --- | --- | --- | --- |
| PPAR14 | 0.149 (0.003 to 3.14) | 0.066 (0.35 to 3.49) | 1 (-1.22 to 1.92) | <0.0001 (1.92 to 3.49) |

**Supplementary Table 5.** Significance (Bonferroni adjusted p-value) and Confidence Interval (C.I.) of hormones and analytes.

| Analyte/ adj p-value (C.I.) | TS vs ST3D | TS vs EVT | ST3D vs EVT | Control vs ZIKV |
| --- | --- | --- | --- | --- |
| CG | <0.001 (3.16 to 5.44) | <0.001 (2.95 to 5.23) | 1 (-1.35 to 0.93) | 0.525 (-0.26 to 0.48) |
| progesterone | 0.002 (0.51 to 1.25) | <0.001 (0.876 to 1.62) | 0.155 (-0.003 to 0.74) | 0.031 (0.03 to 0.52) |
| IL-1RA | <0.001 (1.03 to 2.13) | <0.001 (0.94 to 2.04) | 1 (-0.65 to 0.45) | 0.003 (0.15 to 0.52) |
| bNGF | 0.003 (0.55 to 1.58) | 0.002 (0.62 to 1.65) | 1 (-0.45 to 0.58) | 0.010 (0.08 to 0.44) |
| IL-6 | 0.118 (-0.95 to -0.03) | 1 (-0.26 to 0.66) | 0.023 (0.23 to 1.15) | 0.012 (0.08 to 0.52) |
| FGF2 | 0.002 (0.35 to 0.95) | 1 (-0.28 to 0.32) | 0.003 (-0.93 to -0.33) | 0.007 (0.08 0.39) |
| VEGF-A | 0.017 (0.31 to 1.35) | 0.725 (-0.81 to 0.23) | 0.003 (-1.64 to -0.60) | 0.015 (0.06 to 0.41) |
| VEGF-D | 0.001 (0.49 to 1.20) | 1 (-0.36 to 0.34) | 0.001 (-1.21 to -0.51) | 0.015 (0.07 to 0.45) |
| MMP2 | 0.022 (0.31 to 1.31) | 0.222 (-0.01 to 0.99) | 0.732 (-0.82 to 0.18) | 0.029 (0.14 to 0.67) |
| IL-8 | 1 (-0.68 to 1.03) | 1 (-1.24 to 0.48) | 0.525 (-1.41 to 0.30) | 0.009 (0.12 to 0.63) |
| MPC1 | 1 (-1.06 to 0.41) | 0.204 (-1.41 to 0.06) | 0.930 (-1.09 to 0.39) | 0.008 (0.12 to 0.62) |
| MIP-1BETA | 1 (-0.49 to 0.71) | 0.307 (-1.08 to 0.12) | 0.159 (-1.19 to 0.01) | 0.020 (0.054 to 0.50) |
| INF-beta | 1 (-1.27 to 0.65) | 1 (-1.28 to 0.65) | 1 (-0.97 to 0.95) | 0.066 (-1.43 to 0.06) |
| IL-28a | 0.371 (-0.15 to 1.09) | 0.987 (-0.34 to 0.90) | 1 (-0.80 to 0.44) | 0.066 (-0.03 to 0.82) |

| Analyte/ adj  p-value (C.I.) | TSC-C vs TSC-Z | ST3D-C vs ST3D-Z | EVT-C vs EVT-Z | TSC-C vs ST3D-C | TSC-C vs EVT-C | ST3D-C vs EVT-C | TSC-Z vs ST3D-Z | TSC-Z vs EVT-Z | ST3D-Z vs EVT-Z |
| --- | --- | --- | --- | --- | --- | --- | --- | --- | --- |
| ITAC | 0.428  (0.006 to 0.82) | <0.001  (0.80 to 1.62) | 1  (-0.19 to 0.63) | 1  (-0.21 to 1.14) | 0.642  (-0.06 to 1.29) | 1  (-0.52 to 0.83) | 0.009  (0.58 to 1.93) | 1  (-0.26 to 1.09) | 0.161  (-1.51 to -0.16) |
